# Respiratory chain components are required for peptidoglycan recognition protein-induced thiol depletion and killing in *Bacillus subtilis* and *Escherichia coli*

**DOI:** 10.1038/s41598-020-79811-z

**Published:** 2021-01-08

**Authors:** Chun-Kai Yang, Des R. Kashyap, Dominik A. Kowalczyk, David Z. Rudner, Xindan Wang, Dipika Gupta, Roman Dziarski

**Affiliations:** 1 Indiana University School of Medicine–Northwest, Gary, IN 46408 USA; 2 Department of Microbiology, Harvard Medical School, Boston, MA 02115 USA; 3 Department of Biology, Indiana University, Bloomington, IN 47405 USA

**Keywords:** Bacteriology, Innate immunity

## Abstract

Mammalian peptidoglycan recognition proteins (PGRPs or PGLYRPs) kill bacteria through induction of synergistic oxidative, thiol, and metal stress. Tn-seq screening of *Bacillus subtilis* transposon insertion library revealed that mutants in the shikimate pathway of chorismate synthesis had high survival following PGLYRP4 treatment. Deletion mutants for these genes had decreased amounts of menaquinone (MK), increased resistance to killing, and attenuated depletion of thiols following PGLYRP4 treatment. These effects were reversed by MK or reproduced by inhibiting MK synthesis. Deletion of cytochrome *aa*_*3*_-600 or NADH dehydrogenase (NDH) genes also increased *B. subtilis* resistance to PGLYRP4-induced killing and attenuated thiol depletion. PGLYRP4 treatment also inhibited *B. subtilis* respiration. Similarly in *Escherichia coli*, deletion of ubiquinone (UQ) synthesis, formate dehydrogenases (FDH), NDH-1, or cytochrome *bd*-I genes attenuated PGLYRP4-induced thiol depletion. PGLYRP4-induced low level of cytoplasmic membrane depolarization in *B. subtilis* and *E. coli* was likely not responsible for thiol depletion. Thus, our results show that the respiratory electron transport chain components, cytochrome *aa*_*3*_-600, MK, and NDH in *B. subtilis*, and cytochrome *bd*-I, UQ, FDH-O, and NDH-1 in *E. coli*, are required for both PGLYRP4-induced killing and thiol depletion and indicate conservation of the PGLYRP4-induced thiol depletion and killing mechanisms in Gram-positive and Gram-negative bacteria.

## Introduction

Peptidoglycan Recognition Proteins (PGRPs or PGLYRPs) are evolutionarily conserved from insects to vertebrates and function in antibacterial innate immunity^1,2^. Mammals have four PGRPs (coded by *PGLYRP1-4* genes)—three are bactericidal proteins (PGLYRP1, PGLYRP3, and PGLYRP4)^3–6^ and one is an enzyme, a peptidoglycan amidohydrolase (PGLYRP2)^7,8^. All PGRPs have one or two conserved PGRP domains, which bind bacterial peptidoglycan or its fragments^1,2^.

Bactericidal PGRPs bind to peptidoglycan in Gram-positive bacteria or to the outer membrane in Gram-negative bacteria, but do not enter the cytoplasm and kill bacteria from this extracellular site by simultaneously inducing three synergistic stress responses: oxidative stress, thiol stress, and metal stress^9,10^. Similar bacterial killing can be reproduced by simultaneous treatment of bacteria with chemicals that induce oxidative stress (paraquat), thiol stress (diamide), and metal stress (metals)^10^.

PGRP-induced oxidative stress is due to an increased production of hydrogen peroxide (H_2_O_2_) and hydroxyl radicals (HO^·^) and results in high induction of oxidative stress response genes, including the PerR regulon in *Bacillus subtilis* and OxyR and SoxR regulons in *Escherichia coli*^9,10^. PGRP-induced thiol (disulfide) stress in both *B. subtilis* and *E. coli* is due to depletion (oxidation) of over 90% of cellular thiols and results in high induction of expression of thiol stress response genes, including genes for chaperones and protein quality control^10^. PGRP-induced metal stress is due to increases in intracellular free (labile) Zn^2+^ and Cu^+^ in *B. subtilis* and Zn^2+^ in *E. coli* and results in high increase in the expression of metal efflux and metal detoxification genes^10^. All three stresses (oxidative, thiol, and metal) are required for the PGRP-induced bacterial killing, because inhibition of even one of these stress responses—H_2_O_2_ and HO^·^ production (by anaerobic conditions or dipyridyl), or thiol depletion (by thiourea), or metal stress (by selective chelation of Zn^2+^ or Cu^+^)—abolishes PGRP-induced bacterial killing^9,10^. Each stress response individually is only bacteriostatic, but not bactericidal^9,10^.

Depletion (oxidation) of thiols is often regarded as a consequence of the release of reactive oxygen species and thus as a consequence or a part of oxidative stress^10–16^. Similarly, metal stress (caused by an increase in intracellular concentration of free metal ions) is also often regarded as a consequence of oxidative and thiol stress^10–12,17,18^. However, PGRP-induced oxidative, thiol, and metal stress are mostly independent of each other because: (i) the amount of H_2_O_2_ induced by PGRP does not induce thiol depletion^10^; (ii) thiol depletion induced by an electrophile diamide does not induce H_2_O_2_ and oxidative stress^10^; (iii) several *E. coli* mutants (such as TCA cycle mutants) that are deficient in PGRP-induced H_2_O_2_ production still have efficient PGRP-induced thiol depletion^11^; (iv) PGRPs efficiently induce metal stress in all *E. coli* mutants that are deficient in PGRP-induced H_2_O_2_ production^11^; and (v) H_2_O_2_ production induced by paraquat or thiol depletion induced by diamide, at the levels similar to the levels induced by PGRP, do not induce metal stress^10^. Therefore, each of these stress responses (oxidative, thiol, and metal) could be induced by PGRP through a different mechanism.

It is not known how PGRPs induce thiol depletion and the resultant thiol stress in bacteria. The consequences of thiol stress responses induced by PGRP and diamide are similar^10^. However, diamide is a low molecular weight electrophile that enters the cells and directly abstracts electrons from the thiol groups^12,16^. By contrast, because PGRPs are large proteins that do not enter the cytoplasm^4,9^, they are unlikely to directly function as electrophiles and are more likely to induce thiol depletion indirectly by inducing some, so far unknown, metabolic reactions in bacteria. Therefore, the aim of this study was to begin to elucidate the mechanism of PGRP-induced thiol depletion required for bacterial killing of both Gram-positive and Gram-negative bacteria using *B. subtilis* and *E. coli* as models. Our results reveal that components of the respiratory electron transport chain are required for PGRP (PGLYRP4)-induced thiol stress and killing in both *B. subtilis* and *E. coli*.

## Results

### Tn-seq identifies *B. subtilis* shikimate synthesis pathway mutants with increased survival in PGLYRP4-treated cultures

We used a *B. subtilis* transposon (Tn) insertion library of ~ 138,000 unique mutants with one transposon randomly inserted into the chromosome in each mutant^19,20^. We treated the library for 3 h with bovine serum albumin (BSA) as a control or with a bactericidal concentration of PGLYRP4 that reduced the numbers of colony forming units (CFU) by > 99.5%, or with a sub-bactericidal concentration of PGLYRP4 that reduced CFU by ~ 83%. This 3-h treatment allowed killing and depletion of PGLYRP4-sensitive mutants and selection and enrichments of PGRP-resistant mutants. We used human recombinant PGLYRP4 as a representative bactericidal PGRP because the mechanism of bacterial killing by all mammalian bactericidal PGRPs is similar^4,6,9,10^. We treated bacteria with BSA as a control to account for any possible differences in the frequencies of mutants that could result from their different growth rates during the 3-h treatment. We then identified mutated genes in surviving bacteria using Illumina Mi-Seq and for each gene we calculated the survival index (SI), which is a change in the frequency of each Tn insertion mutant in PGLYRP4-treated compared with control BSA-treated cultures. A mutation with no effect on the survival in the presence of PGLYRP4 compared with BSA has SI = 1. SI > 1 represents a mutation that makes bacteria more resistant to PGLYRP4, which indicates that the product of this gene enhances PGLYRP4 killing. SI < 1 represents a mutation that makes bacteria more sensitive to PGLYRP4, which indicates that the product of this gene protects bacteria from PGLYRP4 killing. We focused on mutants with increased SI to identify genes that may participate in PGLYRP4-induced killing.

**Figure 1:**
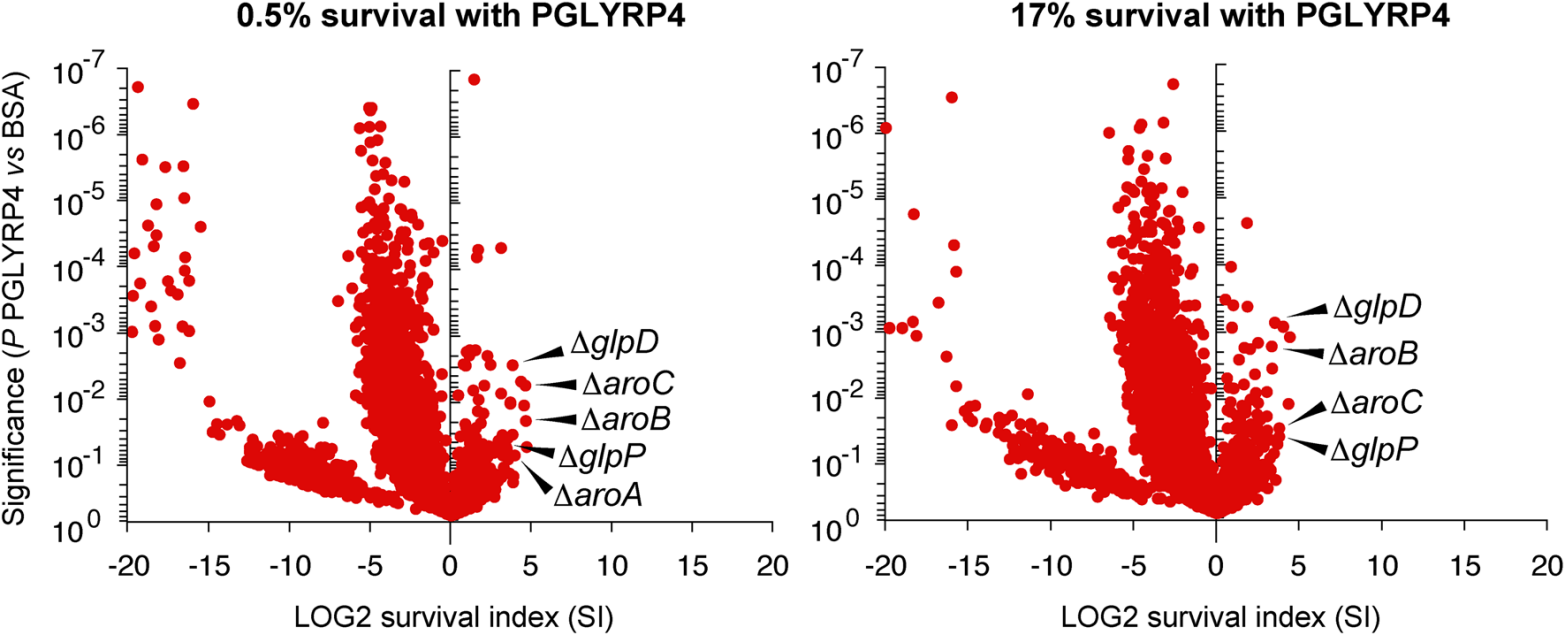
Tn-seq identifies association of shikimate synthesis genes (*aroA*, *aroB*, *aroC*, *glpD*, *glpP*) with PGLYRP4-induced killing in *B. subtilis*. Tn insertion library was treated for 3 h with BSA as a control or with PGLYRP4 at bactericidal (0.5% survival) or sub-bactericidal (17% survival) concentration, and the survival index (SI) with PGLYRP4, relative to BSA, for individual Tn-mutants was determined by Tn-seq. The results are means from 3 independent experiments (biological replicates) and each dot represents a single gene insertion mutant. The Tn insertion sites and the numbers of reads for the *aroA*, *aroB*, *aroC*, *glpD* genes are shown in Supplementary Fig. S1. All Tn-seq mutants with significantly increased frequency in the Tn-seq library in PGLYRP4-treated cultures are shown in Supplementary Tables S1, S2. The complete Tn-seq results have been deposited in NCBI SRA with accession number PRJNA628733.

In cultures treated with bactericidal or sub-bactericidal concentrations of PGLYRP4, 50 and 71 Tn-seq mutants (respectively) had significantly increased SI (*P* ≤ 0.05, Fig. 1, Supplementary Tables S1, S2). These mutated genes had diverse functions, including synthesis (~ 38%), regulation (13–20%), unknown function (~ 18%), transport (8–14%), and redox reactions (8–10%). The complete Tn-seq results have been deposited in NCBI SRA with accession number PRJNA628733.

**Figure 2:**
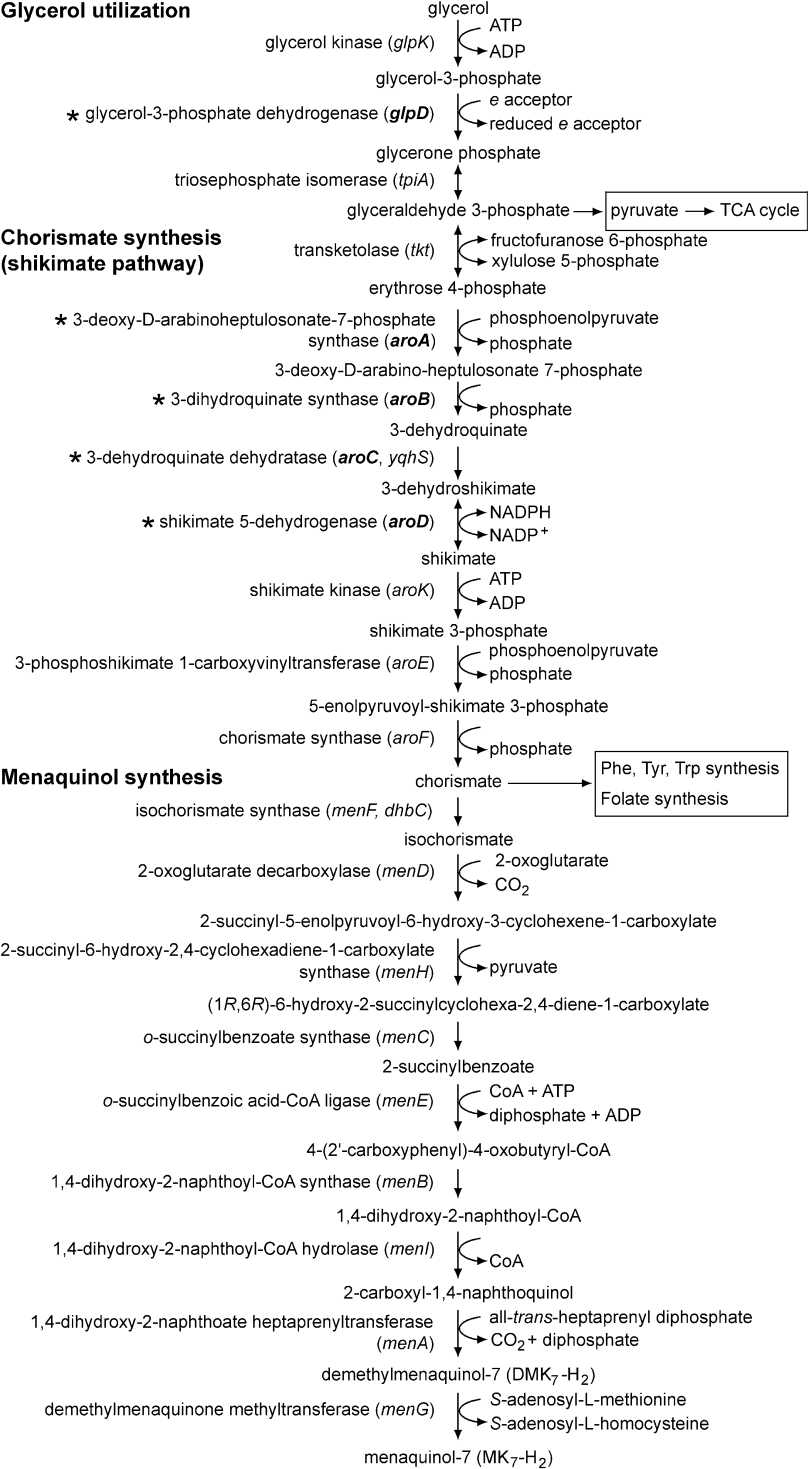
Glycerol utilization and synthesis of shikimate, chorismate, and menaquinol in *B. subtilis* (based on BsubCyc https://bsubcyc.org/^24^ and^21,23^); *aroA-D* and *glpD* are indicated by an asterisk (*).

Two out of the top three and five out of top twelve Tn-seq mutants with the most increased frequency in the Tn-seq library treated with the bactericidal concentration of PGLYRP4 were *aroA*, *aroB*, *aroC*, *glpD*, and *glpP*. Four of these Tn-seq mutants (*aroB*, *aroC*, *glpD*, and *glpP*) were also among the top ten mutants with the most increased frequency in Tn-seq library treated with the sub-bactericidal concentration of PGLYRP4 (Fig. 1, Supplementary Fig. S1, Supplementary Tables S1, S2). These genes code for 3-deoxy-d-arabinoheptulosonate-7-phosphate synthase (*aroA*), 3-dihydroquinate synthase (*aroB*), and 3-dehydroquinate dehydratase (*aroC*) in the shikimate pathway for the synthesis of chorismate, and glycerol-3-phosphate dehydrogenase (*glpD*) and activator of *glpD* transcription (*glpP*) in the glycerol utilization pathway (Fig. 2) (BsubCyc https://bsubcyc.org/)^21–24^. In this study we further pursued the role of these *aro* and *glp* genes in PGLYRP4-induced killing and thiol stress because: (i) these genes were consistently and most highly enriched in cultures treated with both concentrations of PGLYRP4; (ii) they could be placed in one biosynthetic pathway involved in the utilization of glycerol and synthesis of demethylmenaquinone (DMK) and menaquinone (MK), in addition to other pathways (Fig. 2)^21–25^; (iii) MK and DMK are components of *B. subtilis* respiratory electron transport chain and electrophiles that could be involved in thiol depletion; and (iv) ubiquinone and electron transport chain are involved in PGLYRP4-induced killing and oxidative stress in *E. coli*^11,26^, and, thus, quinones could also play a role in PGLYRP4-induced thiol stress.

### *B. subtilis* shikimate pathway genes are required for PGLYRP4-induced killing through their effect on menaquinone synthesis

**Figure 3:**
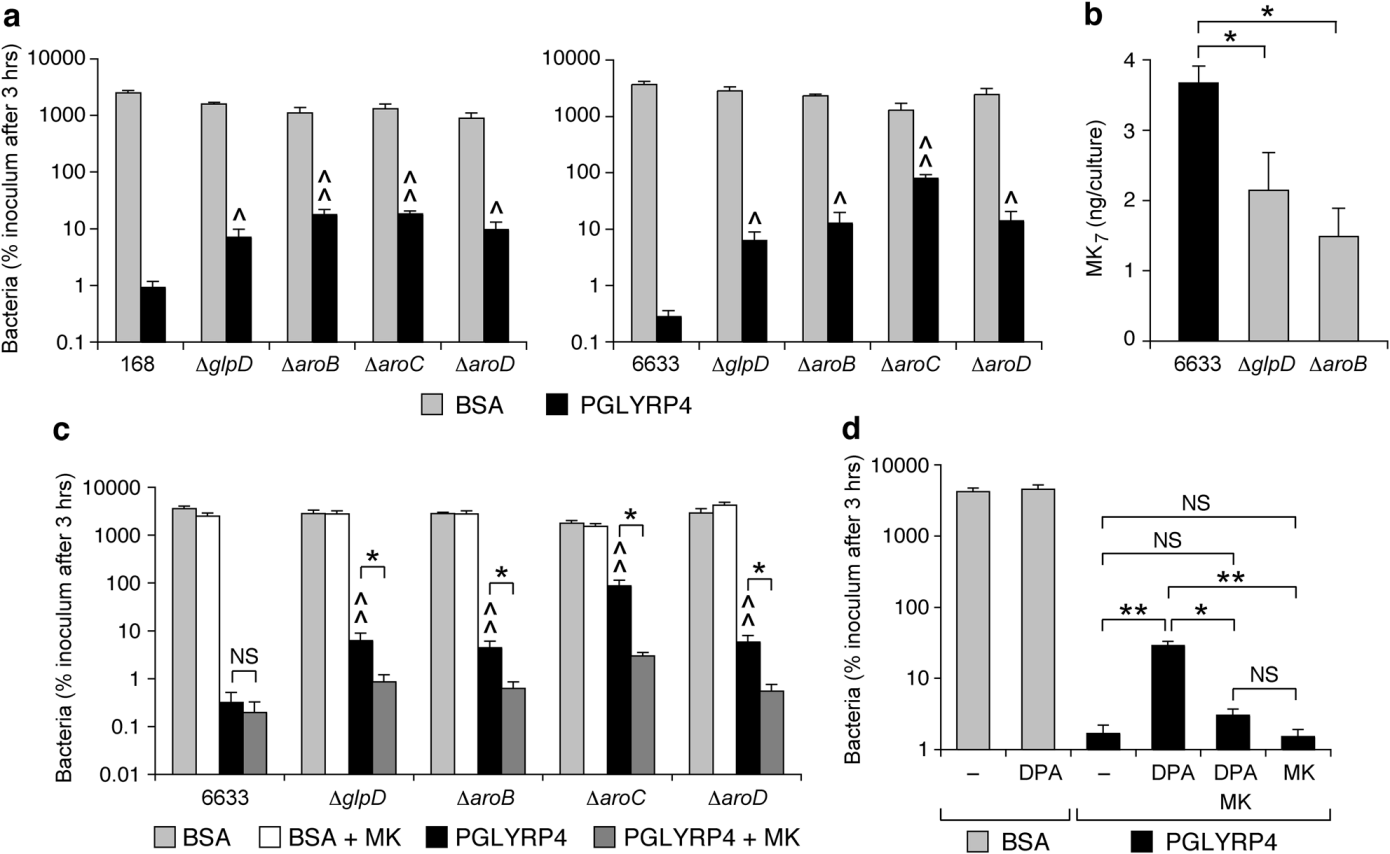
*B. subtilis* Δ*glpD* and Δ*aro* mutants have increased resistance to PGLYRP4-induced killing (**a**), lower amounts of menaquinone (MK) (**b**), exogenous MK increases their sensitivity to PGLYRP4-induced killing (**c**), and MK synthesis inhibitor DPA inhibits PGLYRP4-induced killing, which is restored by addition of exogenous MK (**d**). The results are means ± SEM of the numbers of bacteria (CFU) expressed as percent of initial inoculum (100%) (**a**,**c**,**d**) or of amounts of MK_7_/culture (**b**) and are from 4 to 6 (**a**,**c**,**d**) or 3 (**b**) experiments (biological replicates). *B. subtilis* 6633 was used in (**d**). ^^^*P* < 0.05, ^^^^*P* < 0.001 mutant *vs* parental strain; *NS* not significant (*P* > 0.05); *P < 0.05, **P < 0.001 as indicated by brackets.

Increased frequency of Δ*aroB*, Δ*aroC*, Δ*aroD*, Δ*glpD*, and Δ*glpP* Tn mutants in PGLYRP4-treated cultures suggested that these genes may be required for PGLYRP4-induced killing of *B. subtilis* and that this biosynthetic pathway may participate in PGLYRP4-induced killing. To test this hypothesis, we tested the sensitivity of deletion mutants for these genes in *B. subtilis* 168 strain^27^. Moreover, to further verify the role of these genes we constructed these deletion mutants in another strain, *B. subtilis* 6633, and also tested their sensitivity to PGLYRP4-induced killing. Δ*glpD*, Δ*aroB*, Δ*aroC*, and Δ*aroD* mutants in both *B. subtilis* 168 and 6633 were significantly more resistant to PGLYRP4-induced killing than their parental strains, with 8- and 22-, 11- and 45-, 19- and 285-, or 20- and 50-fold higher numbers of CFU than in the parental 168 and 6633 strains, respectively (Fig. 3a).

GlpD has several functions, as its product, glycerone phosphate (dehydroxyacetone phosphate), after isomerization to glyceraldehyde 3-phosphate, can be used to synthesize pyruvate for the TCA cycle and other reactions, in addition to entering the shikimate pathway of chorismate synthesis (Fig. 2) (BsubCyc https://bsubcyc.org/)^21–25^. In *E. coli* GlpD serves as a dehydrogenase (quinone reductase) in the cytoplasmic membrane electron transport chain^25^ and in *B. subtilis* GlpD could play a similar role. Chorismate is the substrate for the synthesis of DMK and MK, but it is also used in other biosynthetic pathways, such as phenylalanine, tyrosine, tryptophan, and folate synthesis (Fig. 2) (BsubCyc https://bsubcyc.org/)^23,24^. Thus, we then tested the hypothesis that DMK and MK may be required for PGLYRP4-induced killing of *B. subtilis*, because in *E. coli* ubiquinone and other components of the respiratory electron transport chain are required for PGLYRP4-induced killing^11,26^. However, in contrast to *E. coli*^26^, the role of DMK and MK in PGLYRP4-induced killing cannot be directly tested using DMK and MK deletion mutants because MK and DKM are the only quinones in *B. subtilis* and are essential for *B. subtilis* survival in media without exogenously added MK or DMK. Thus, deleting the *men* genes for MK and DMK synthesis (Fig. 2) generates mutants that do not grow in the absence of exogenous MK or DMK. For this reason we used three approaches to determine whether the effect of deletions of *glpD*, *aroB*, *aroC*, and *aroD* on PGLYRP4-induced killing of *B. subtilis* was due to the impairment of MK and DMK synthesis or due to the impairment of other metabolic reactions that utilize glyceraldehyde 3-phosphate or chorismate.

As the first approach, we tested whether *glpD* and *aro* deletion mutants have decreased amounts of MK. Indeed, *B. subtilis* 6633 Δ*glpD* and Δ*aroB* deletion mutants had significantly lower amounts of MK than the parental strain (58% and 40% of the parenteral strain, respectively) (Fig. 3b).

As the second approach, we tested whether adding exogenous MK to the cultures of Δ*glpD*, Δ*aroB*, Δ*aroC*, and Δ*aroD* deletion mutants would reverse their resistance to PGLYRP4 killing, which would be an indication that the effect of these deletions is indeed through their inhibitory effect on MK synthesis. As predicted, adding exogenous MK to the cultures of Δ*glpD*, Δ*aroB*, Δ*aroC*, and Δ*aroD* deletion mutants significantly reversed their resistance to PGLYRP4-induced killing to the level comparable with the killing of the parental strain, whereas exogenous MK had no significant effect on PGLYRP4-induced killing of the parental strain or on the growth of control BSA-treated parental or mutant strains (Fig. 3c).

As the third approach, we tested whether diphenylamine (DPA), an inhibitor of MK synthesis^28,29^, would increase resistance of *B. subtilis* to PGLYRP4-induced killing, and also whether supplementing the medium with exogenous MK would reverse the resistance to PGLYRP4 killing (to verify the selectivity of DPA for inhibition of MK synthesis). Indeed, DPA significantly increased the resistance of *B. subtilis* to PGLYRP4-induced killing and adding exogenous MK reversed this effect, whereas DPA did not affect the growth of control BSA-treated bacteria (Fig. 3d).

Altogether, these results indicate that the increased resistance of *B. subtilis* Δ*glpD*, Δ*aroB*, Δ*aroC*, and Δ*aroD* deletion mutants to PGLYRP4-induced killing is likely due to the effect of these deletions on MK synthesis and suggest that MK and/or DMK synthesis through the shikimate pathway is required for efficient PGLYRP4-induced killing. These results also suggest alternative pathway(s) of chorismate synthesis (in addition to the shikimate pathway) because shikimate pathway mutants still contain low amounts of MK and are viable without addition of exogenous MK. We verified the expression of *aroB*, *aroC*, *aroD*, and *glpD* under our culture conditions (Supplementary Fig. S2).

### *B. subtilis* shikimate pathway genes are required for PGLYRP4-induced thiol depletion through their effect on menaquinone synthesis

**Figure 4:**
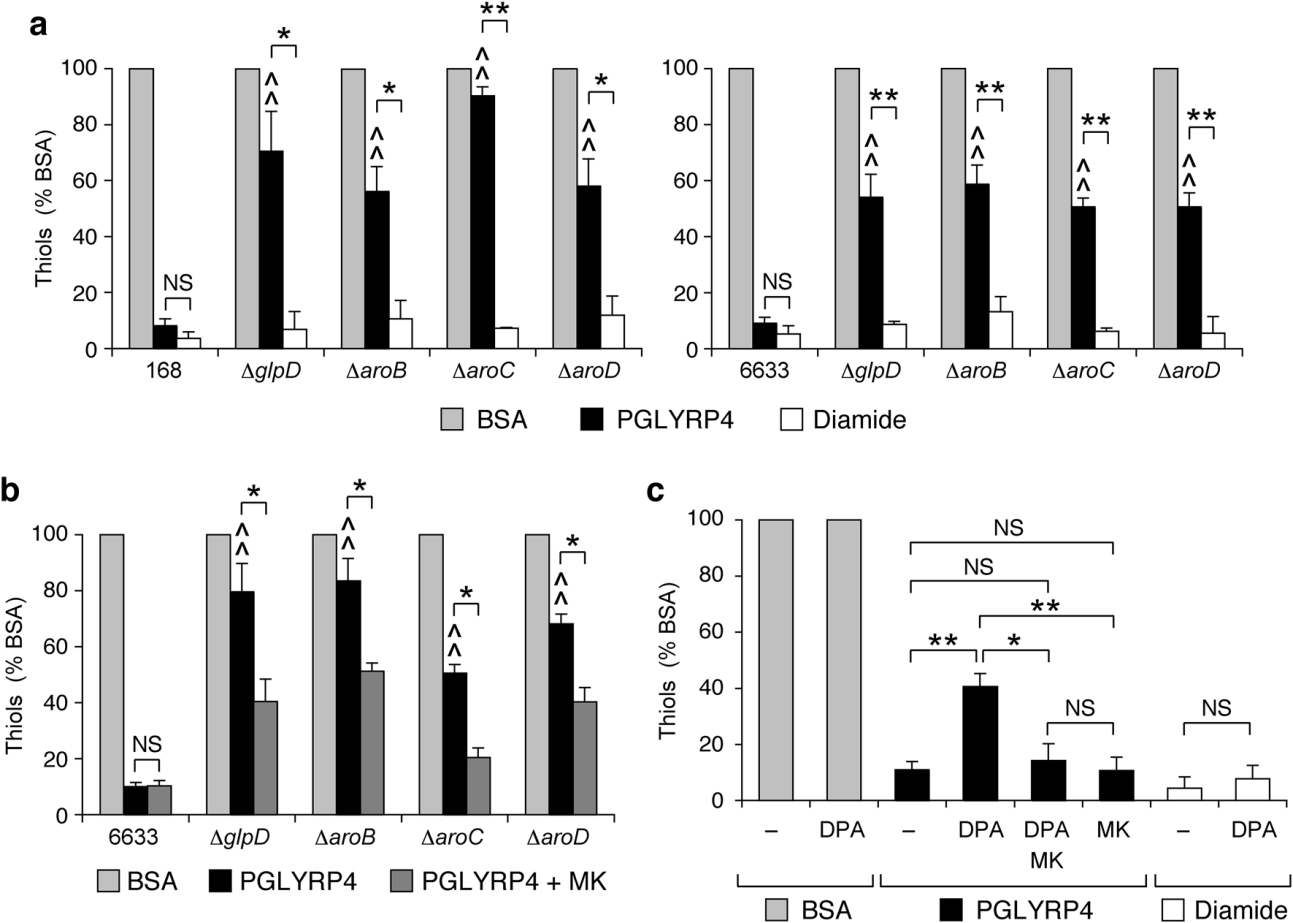
*B. subtilis* Δ*glpD* and Δ*aro* mutants have attenuated PGLYRP4-induced depletion of thiols (**a**), exogenous menaquinone (MK) increases PGLYRP4-induced depletion of thiols in these mutants (**b**), and MK synthesis inhibitor DPA attenuates PGLYRP4-induced depletion of thiols, which is restored by addition of exogenous MK (**c**). The results are the amounts of thiols expressed as percent of control with BSA (100%) and are means ± SEM from 4 to 6 experiments (biological replicates). *B. subtilis* 6633 was used in (**c**). ^^^*P* < 0.05, ^^^^*P* < 0.001 mutant *vs* parental strain; *NS* not significant (*P* > 0.05); *P < 0.05, **P < 0.001 as indicated by brackets.

We next tested the effect of deletions of *glpD*, *aroB*, *aroC*, and *aroD* genes on the PGLYRP4-induced thiol depletion because this severe depletion of cellular thiols (thiols oxidation) is required for PGRP-induced killing of *B. subtilis*^10^. In parental *B. subtilis* 168 and 6633 strains PGLYRP4 induced depletion of 95–96% cellular thiols, whereas Δ*glpD*, Δ*aroB*, Δ*aroC*, and Δ*aroD* deletion mutants in both *B. subtilis* 168 and 6633 strains had significantly attenuated PGLYRP4-induced thiol depletion (10–42% and 41–49% depletion, respectively). By contrast, thiol depletion induced by diamide, an electrophile that directly oxidizes thiols^12,16^, was similar in both parental strains and all deletion mutants (87–96%) (Fig. 4a).

We then tested whether the attenuated PGLYRP4-induced thiol depletion in Δ*glpD*, Δ*aroB*, Δ*aroC*, and Δ*aroD* deletion mutants was related to the participation of these genes in MK and DMK synthesis, because this was the case in the PGLYRP4-induced killing and because oxidized MK and DMK are electrophiles that could play a role in thiol depletion. Adding exogenous MK to the cultures of Δ*glpD*, Δ*aroB*, Δ*aroC*, and Δ*aroD* deletion mutants significantly restored PGLYRP4-induced thiol depletion but had no effect on already efficient thiol depletion in the parental strain (Fig. 4b). Moreover, MK synthesis inhibitor DPA significantly attenuated PGLYRP4-induced thiol depletion and adding exogenous MK reversed this effect, whereas DPA did not affect diamide-induced thiol depletion or the thiol level in control BSA-treated bacteria (Fig. 4c).

Altogether, these results indicate that the attenuated PGLYRP4-induced thiol depletion in *B. subtilis* Δ*glpD*, Δ*aroB*, Δ*aroC*, and Δ*aroD* deletion mutants is likely due to the effect of these deletions on MK synthesis. These results suggest that MK and/or DMK synthesis through the shikimate pathway is required for efficient PGLYRP4-induced thiol depletion, in contrast to the diamide-induced thiol depletion, which does not rely on the shikimate pathway and MK, consistent with the direct oxidation of thiols by diamide^12,16^.

Our results on the involvement of *glpD* in MK synthesis are consistent with the recent results showing a prominent role of *glpD* in MK synthesis in *B. subtilis* because overexpression of *glpD* increased the synthesis of MK^23^. The previously mentioned additional functions of *glpD* seem unlikely to play a role in PGLYRP4-induced killing and thiol depletion because they would not be reversed by exogenously added MK in Δ*glpD* mutants or inhibited by an MK synthesis inhibitor. In this study we did not further pursue these other functions of *glpD* and focused on the role of the respiratory chain in PGLYRP4-induced killing and thiol depletion.

### ***B. subtilis*** NADH dehydrogenase and cytochrome ***aa***_***3***_-600 are required for PGLYRP4-induced killing and thiol depletion

We next tested the role of the respiratory electron transport chain components, NADH dehydrogenase (NDH) and cytochromes, in PGLYRP4-induced killing and thiol depletion in *B. subtilis* because MK and DMK are an integral part of this electron transport chain, as they accept electrons from NADH and then donate these electrons to the cytochromes (BsubCyc https://bsubcyc.org/)^24,30,31^.

**Figure 5:**
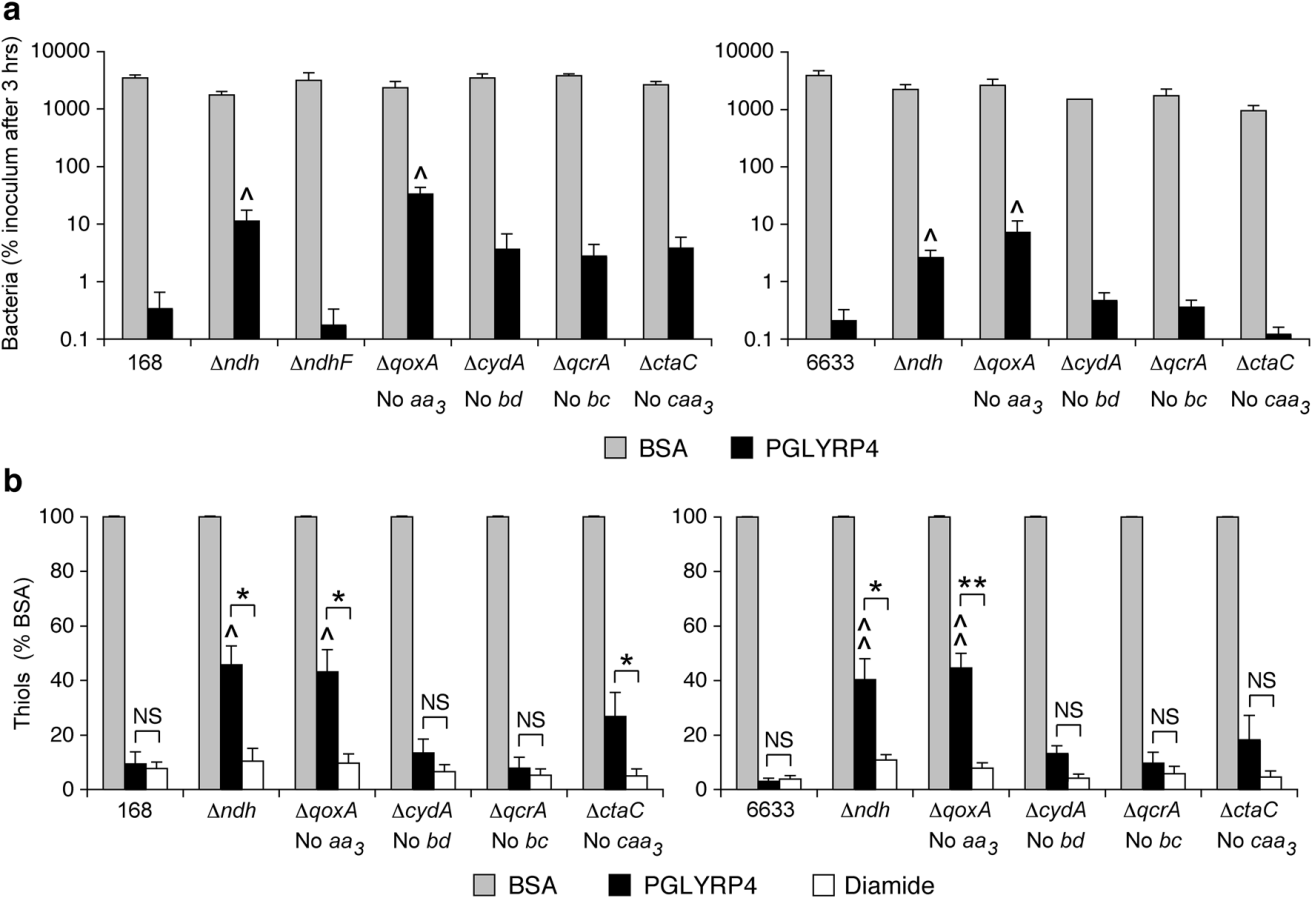
Deletion of genes for NDH (Δ*ndh*) or cytochrome *aa*_*3*_-600 (Δ*qoxA*) increases resistance of *B. subtilis* to PGLYRP4-induced killing and attenuates PGLYRP4-induced thiol depletion. The results are the numbers of bacteria (CFU) expressed as percent of initial inoculum (100%) (**a**) or the amounts of thiols expressed as percent of control with BSA (100%) (**b**) and are means ± SEM from 4 to 6 experiments (biological replicates); ^^^*P* < 0.05, ^^^^*P* < 0.001 mutant *vs* parental strain; *NS* not significant (*P* > 0.05); *P < 0.05, **P < 0.001 as indicated by brackets.

*B. subtilis* has two NDH genes: *ndh* and *ndhF*, which code for the proven and putative NADH dehydrogenases, respectively (BsubCyc https://bsubcyc.org/)^24^. Δ*ndh* mutants in both *B. subtilis* 168 and 6633 strains were significantly more resistant to PGLYRP4-induced killing (with 34- and 13-fold higher numbers of CFU than in the parental 168 and 6633 strains, respectively, Fig. 5a). These Δ*ndh* mutants also had significantly attenuated PGLYRP4-induced thiol depletion compared with the parental strains (40–46% depletion compared with 91–97% in the parental strains, Fig. 5b). We verified high expression of *ndh* under our culture conditions (Supplementary Fig. S2). Altogether our results indicate that *ndh* is required for efficient PGLYRP4-induced killing and thiol depletion. By contrast, Δ*ndhF* mutant in *B. subtilis* 168 had similar sensitivity to PGLYRP4-induced killing as the parental strain (Fig. 5a) and for this reason was not used in the subsequent experiments.

*B. subtilis* has a respiratory electron transport chain with two cytochromes, *aa*_*3*_-600 and *bd* (coded by *qoxABCD* and *cydAB*), which accept electrons directly from MK and function as terminal O_2_ reductases. *B. subtilis* also has a branch of the electron transport chain with cytochrome *bc* (coded by *qcrABC*), which accepts electrons from MK and transfers them to intermediate *c*-type cytochromes (coded by *cccA* and *cccB*), which then transfer these electrons to the third terminal O_2_ reductase, cytochrome *caa*_*3*_ (coded by *ctaCDEFG*) (BsubCyc https://bsubcyc.org/)^24,30–32^.

Δ*qoxA* deletion mutant (lacking cytochrome *aa*_*3*_-600) in both *B. subtilis* 168 and 6633 strains had significantly increased resistance to PGLYRP4-induced killing (with 99- and 35-fold higher numbers of CFU than in the parental 168 and 6633 strains, respectively, Fig. 5a), and also had attenuated PGLYRP4-induced thiol depletion compared with the parental strains (55–57% depletion compared with 91–97% in the parental strains, Fig. 5b). By contrast, the remaining cytochrome mutants Δ*cydA*, Δ*qcrA*, and Δ*ctaC* (lacking cytochromes *bd*, *bc*, or *caa*_*3*_, respectively) did not show significantly changed sensitivity to PGLYRP4-induced killing and thiol depletion compared with the parental strains (Fig. 5a,b). We verified high expression of cytochrome *aa*_*3*_-600 (in contrast to lower expression of other cytochromes) under our culture conditions (Supplementary Fig. S2). Altogether, our results indicate that cytochrome *aa*_*3*_-600 is required for efficient PGLYRP4-induced killing and thiol depletion.

By contrast to PGLYRP4, diamide-induced thiol depletion was similar in all the respiratory electron transport chain mutants and parental strains (Fig. 5b), indicating that it does not require NDH and cytochromes, consistent with direct oxidation of thiols by diamide^12,16^ and no requirement for MK.

Altogether, these results indicate that in *B. subtilis* the respiratory electron transport chain components, NDH, MK, DMK, and cytochrome *aa*_*3*_-600, are required for efficient PGLYRP4-induced killing and thiol depletion. Deletion of MK and DMK synthesis genes caused the greatest attenuation of PGLYRP4-induced thiol depletion (change from 93 ± 1.4 to 36 ± 6.2% depletion, *P* < 0.001), followed by NDH and cytochrome *aa*_*3*_-600 (change from 93 ± 1.4 to 57 ± 2.8% and 56 ± 1.0% depletion, respectively; both *P* < 0.001). This reversal of thiol depletion was statistically significant but not complete, likely because of alternative mechanisms still causing some thiol depletion in the mutants, such as alternative pathway(s) of MK and DMK (shikimate and chorismate) synthesis and the effects of other dehydrogenases and cytochromes or other thiol depletion mechanisms.

### PGLYRP4-induces a block in respiration in *B. subtilis*

**Figure 6:**
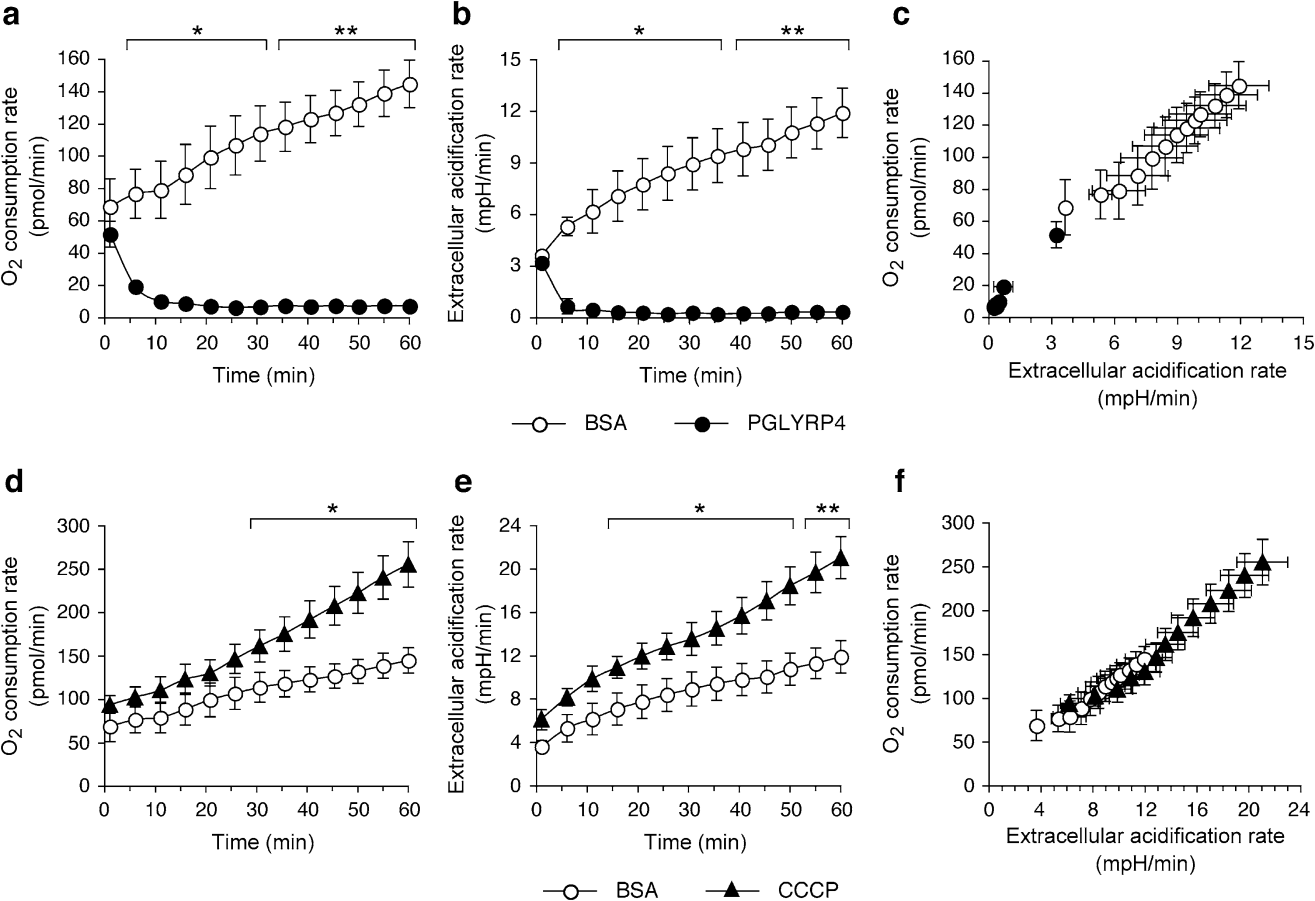
Oxygen consumption and extracellular acidification rates in PGLYRP4- or CCCP-treated *B. subtilis*. (**a**,**d**) O_2_ consumption rate and (**b**,**e**) extracellular acidification rate were measured simultaneously in real time with the Seahorse XFp analyzer in *B. subtilis* 6633 treated with 100 µg/ml BSA, or 100 µg/ml PGLYRP4, or 1 µM CCCP. (**c**,**f**) Phenograms of O_2_ consumption rate plotted *versus* extracellular acidification rate illustrate the total cell energy phenotype. The results are means of 3 experiments ± SEM (SEM were within symbols if not visible); *P < 0.05, **P < 0.001, PGLYRP4 or CCCP *vs* BSA.

Our results presented above suggested that PGLYRP4 treatment of *B. subtilis* caused a malfunction of its aerobic respiratory electron transport chain. We tested this hypothesis directly by measuring in real time PGLYRP4-induced changes in O_2_ consumption rate. We used the Seahorse XFp analyzer, which relies on label-free solid-state sensor cartridges in a microplate format^11,33^. Treatment of *B. subtilis* with PGLYRP4 induced a rapid decrease in O_2_ consumption rate, which was already significantly lower at 6 min and decreased to near baseline level in 20 min, whereas BSA-treated control cells gradually increased O_2_ consumption rate throughout the entire 60-min incubation period, which reflected healthy cell growth (Fig. 6a). As an additional positive control, we used CCCP (carbonyl cyanide 3-chlorophenylhydrazone), a proton ionophore that dissipates both membrane potential and proton gradient, which at low concentrations only partially depolarizes cytoplasmic membrane and stimulates respiration, as the cells attempt to overcome the decrease in membrane potential^33^. As expected, 1 µM CCCP induced a gradual increase in O_2_ consumption rate, which was significantly higher than the BSA-treated group at 30 to 60 min and than the PGLYRP4-treated group at 6 to 60 min (Fig. 6d).

Simultaneously with O_2_ consumption rate, we also measured in real time the extracellular acidification rate (ECAR), which reflects total H^+^ production. ECAR rapidly declined in PGLYRP4-treated *B. subtilis* cells (similar to the decreasing O_2_ consumption rate) and was significantly lower and almost at the baseline already after 6 min of PGLYRP4 treatment (Fig. 6b). By contrast ECAR steadily increased in BSA-treated *B. subtilis* cells throughout the entire 60-min incubation period, which reflected healthy cell growth and was significantly higher than in PGLYRP4-treated cells at 6 to 60 min (Fig. 6b). As expected, ECAR was also significantly higher in CCCP-treated cells than in BSA-treated cells at 16 to 60 min, consistent with higher respiration rate in CCCP- than in BSA-treated cells (Fig. 6e). Plotting O_2_ consumption rate *versus* ECAR illustrates the total cell energy phenotype and shows low-energy phenotype in PGLYRP4-treated cells (Fig. 6c), consistent with eventual energy depletion and impending death^9^, in contrast to the control cells incubated with BSA or CCCP, which had high-energy state (respiration and metabolism) (Fig. 6c,f).

These results show that PGLYRP4 induces a malfunction and eventual shutdown of the respiratory chain in *B. subtilis*. We obtained similar results (decreased O_2_ consumption rate and ECAR) in PGLYRP4-treated *E. coli*^11^, which suggests that the effect of PGLYRP4 on respiration is conserved in Gram-positive and Gram-negative bacteria.

### *E. coli *ubiquinone, cytochrome *bd*-I, and formate and NADH dehydrogenases are required for PGLYRP4-induced thiol depletion

The above results motivated us to investigate whether the respiratory electron transport chain also plays a role in PGLYRP4-induced thiol depletion in Gram-negative bacteria, using *E. coli* as a model. *E. coli* has a complex respiratory electron transport chain that contains 15 dehydrogenases that can deliver electrons to 3 quinones, and 14 terminal reductases that can deliver electrons to 8 different electron acceptors, including 3 terminal cytochromes (*bo*_3_, *bd*-I, and *bd*-II)^25,34^. We have already recently shown that in *E. coli* both NADH dehydrogenases (NDH-1 and NDH-2), formate dehydrogenase-O (FDH-O), ubiquinone (UQ), and cytochrome *bd*-I are all required for PGLYRP4-induced killing and oxidative stress^11,26^, but the role of the respiratory chain components in PGRP-induced thiol stress in *E. coli* was unknown and was the subject of our next experiments.

**Figure 7:**
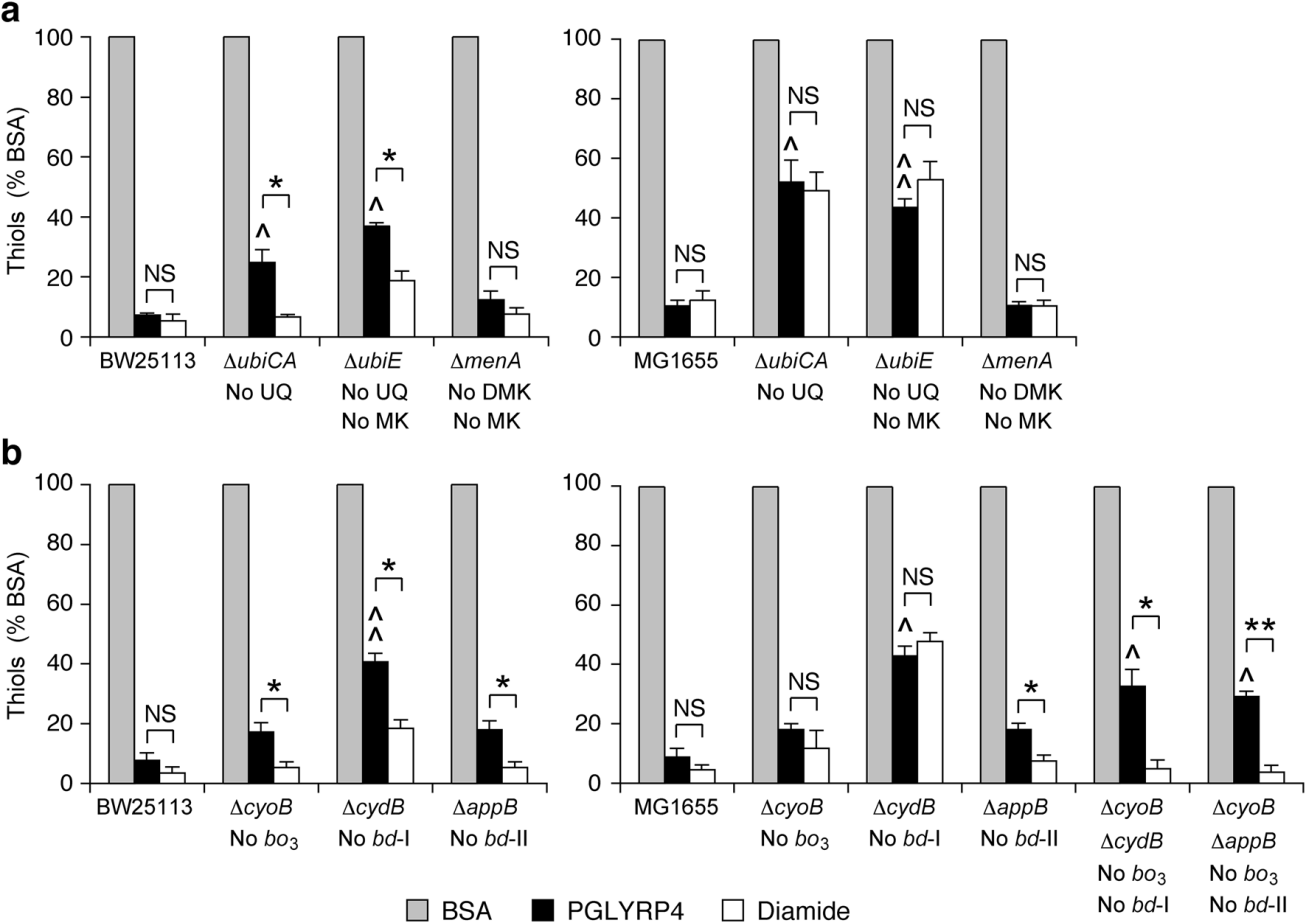
Deletion of ubiquinone (UQ) or cytochrome *bd*-I in *E. coli* attenuates PGLYRP4-induced thiol depletion. The results are the amounts of thiols expressed as percent of control with BSA (100%) and are means ± SEM from 3 experiments (biological replicates); ^^^*P* < 0.05, ^^^^*P* < 0.001 mutant *vs* parental strain; *NS* not significant (*P* > 0.05); *P < 0.05, **P < 0.001 as indicated by brackets.

Given the prominent role of MK and cytochrome *aa*_*3*_-600 in PGLYRP4-induced killing and thiol depletion in *B. subtilis*, we first tested whether quinones and cytochromes were involved in PGLYRP4-induced thiol depletion in *E. coli*. *E. coli* has 3 quinones: UQ, MK, and DMK and we next tested whether any of these quinones are involved in PGLYRP4-induced thiol depletion. In *E. coli* UQ is synthesized by a separate pathway than MK and DMK, but the synthesis of MK from DMK uses the same enzyme (UbiE) that is also used in the synthesis of UQ^35^. Thus, mutants lacking only UQ (Δ*ubiCA*), both UQ and MK (Δ*ubiE*), or both MK and DMK (Δ*menA*) were used. *E. coli* mutants deficient in the synthesis of UQ (Δ*ubiCA*) or UQ and MK (Δ*ubiE*), but not MK and DMK (Δ*menA*), in both BW25113 and MG1655 strains had significantly attenuated PGLYRP4-induced thiol depletion (48–75% depletion) compared with 90–93% thiol depletion in the parental strains (Fig. 7a). Also *E. coli* mutants lacking cytochrome *bd*-I (Δ*cydB*) in both BW25113 and MG1655 strains, but not mutants lacking cytochrome *bo*_3_ (Δ*cyoB*) or cytochrome *bd*-II (Δ*appB*), had significantly attenuated PGLYRP4-induced thiol depletion (57–59% depletion) compared with 91–92% thiol depletion in the parental strains (Fig. 7b). These results show that UQ and cytochrome *bd*-I (but not MK, DMK, and cytochromes *bo*_3_ and *bd*-II) are required for efficient PGLYRP4-induced thiol depletion and are consistent with their requirement for PGLYRP4-induced killing of *E. coli*^26^.

**Figure 8:**
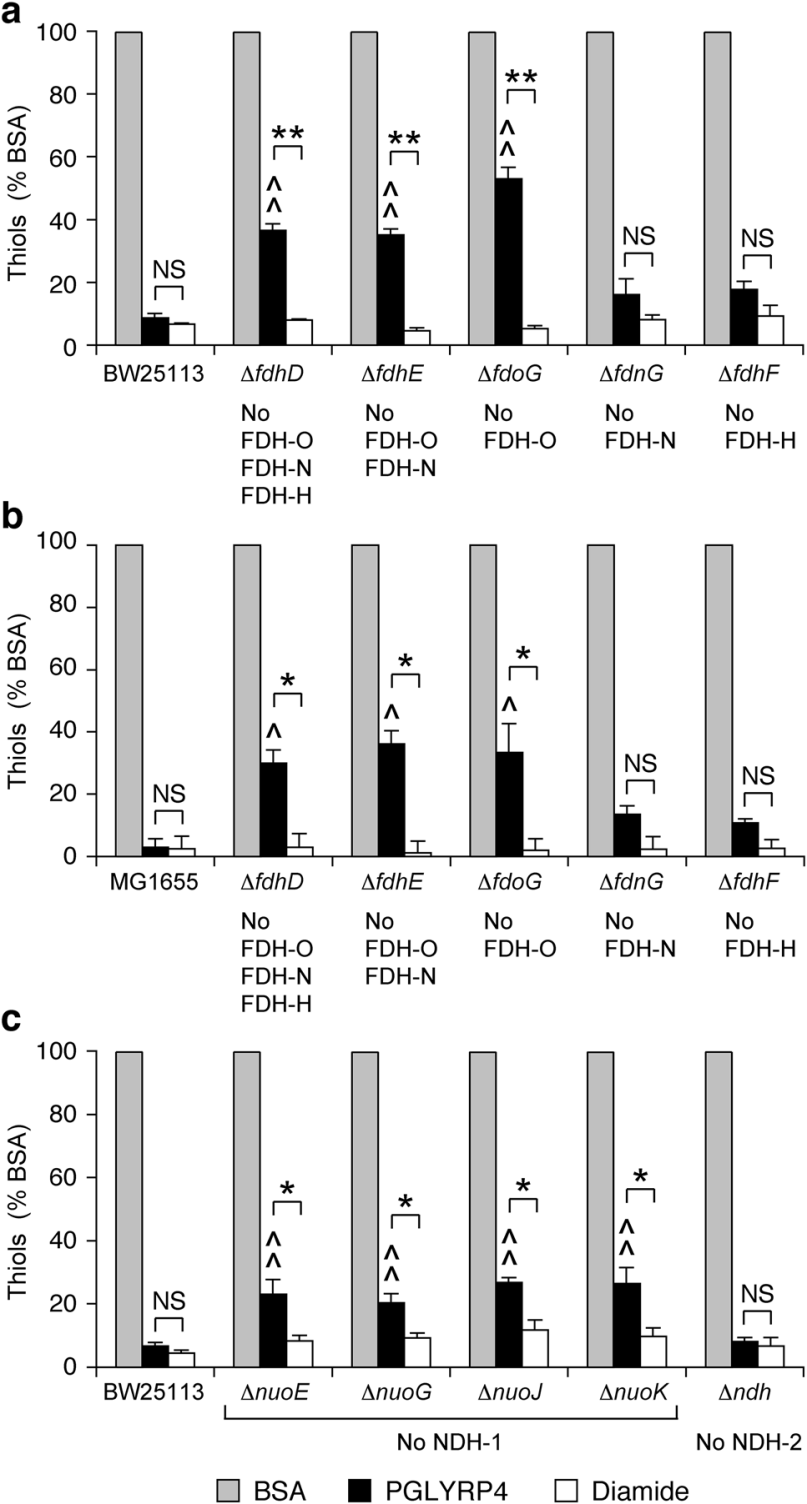
Deletion of FDH or NDH-1 in *E. coli* attenuates PGLYRP4-induced thiol depletion. The results are the amounts of thiols expressed as percent of control with BSA (100%) and are means ± SEM from 3 to 4 experiments (biological replicates); ^^^*P* < 0.05, ^^^^*P* < 0.001 mutant *vs* parental strain; *NS* not significant (*P* > 0.05); *P < 0.05, **P < 0.001 as indicated by brackets. FDH-O, FDH-N, and FDH-H—formate dehydrogenase O, N, and H (respectively); NDH-1 and NDH-2—NADH dehydrogenase I and II (respectively).

We next tested the requirement for the respiratory electron transport chain dehydrogenases in PGLYRP4-induced thiol depletion in *E. coli*, because UQ acquires electrons from these up-stream dehydrogenases and because we have previously shown their requirement for PGLYRP4-induced killing and oxidative stress^11,26^. *E. coli* mutants lacking all three formate dehydrogenases FDH-O, FDH-N, and FDH-H (Δ*fdhD*), or FDH-O and FDH-N (Δ*fdhE*), or FDH-O (Δ*fdoG*) in both BW25113 and MG1655 strains, but not mutants lacking FDH-N (Δ*fdnG*) or FDH-H (Δ*fdhF*), had significantly attenuated PGLYRP4-induced thiol depletion (47–70% depletion) compared with 91–97% thiol depletion in the parental strains (Fig. 8a,b). Also, *E. coli* Δ*nuoE*, Δ*nuoG*, Δ*nuoJ*, and Δ*nuoK* mutants lacking NADH dehydrogenase I (NDH-1), but not mutants lacking NDH-2 (Δ*ndh*), had significantly attenuated PGLYRP4-induced thiol depletion (73–79% depletion) compared with 95% thiol depletion in the parental strain (Fig. 8c). These results are consistent with the requirement of UQ, FDH-O, and NDH-1 for PGLYRP4-induced killing of *E. coli*^11,26^.

By contrast to PGLYRP4, diamide-induced thiol depletion was similar in almost all of the above respiratory electron transport chain mutants (lacking FDH-O, FDH-N, FDH-H, NDH-1, NDH-2, UQ, or cytochrome *bd*-I) and parental strains in *E. coli* (Figs. 7, 8), with the exception of UQ-deficient and cytochrome *bd*-I-deficient mutants only in MG1665, but not in BW25113, background. However, in double deletion Δ*cyoB*Δ*cydB* MG1665 mutant, thiol depletion was only attenuated in PGLYRP4-treated, but not in diamide-treated cells, as expected (Fig. 7b). Therefore, in general, these results indicate selective attenuation of thiol depletion in PGLYRP4-treated cells compared with diamide and we have no explanation for the aberrant results with diamide in these two types of MG1655 mutants (and the reason for these two aberrant responses to diamide was not further pursued in this study, which is focused on the effects of PGLYRP4).

Altogether, our results indicate that efficient PGLYRP4-induced thiol depletion in *E. coli* requires FDH-O, NDH-1, UQ, and cytochrome *bd*-I. Deletion of UQ synthesis and cytochrome *bd*-I genes caused the greatest attenuation of PGLYRP4-induced thiol depletion (change from 93 ± 1.1% to 60 ± 5.1 and 61 ± 2.7% depletion, respectively; both *P* < 0.001), followed by FDH-O and NDH (change from 93 ± 1.1 to 63 ± 3.6% and 76 ± 1.3% depletion, respectively; both *P* < 0.001). The reversal of thiol depletion was statistically significant but not complete, likely because of alterative mechanisms still causing some thiol depletion in the mutants, such as the presence of MK and DMK, the function of other dehydrogenases and cytochromes, or other thiol depletion mechanisms. These results are consistent with the previously shown requirement for these respiratory electron transport chain components in PGLYRP4-induced killing and oxidative stress and with their expression in aerobically growing *E. coli*^11,26^.

### Role of membrane depolarization

**Figure 9:**
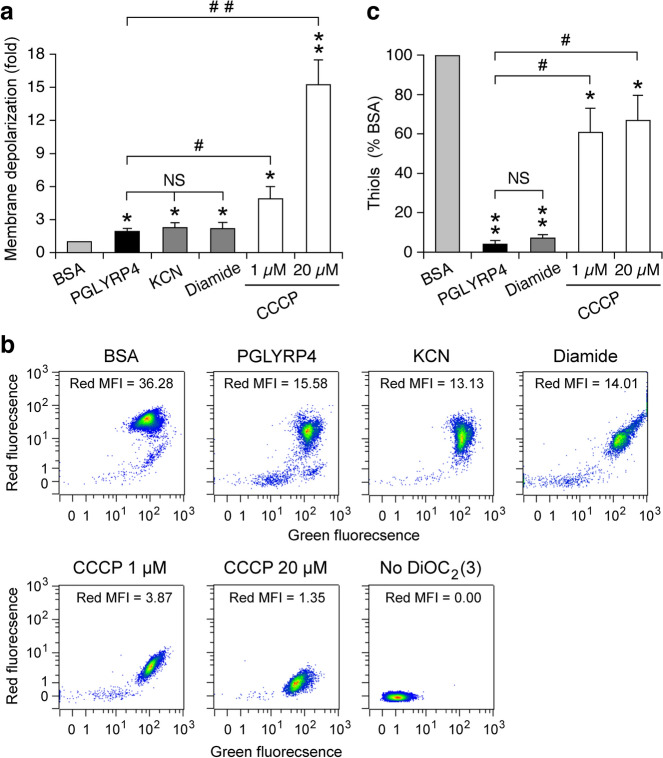
Comparison of membrane depolarization and thiol depletion in *B. subtilis*. *B. subtilis* 168 was treated with BSA, PGLYRP4, KCN, diamide, or CCCP as indicated for 30 min. (**a**) Membrane depolarization was measured by flow cytometry with membrane potential sensitive dye DiOC_2_(3) and the results are the ratios of mean red fluorescence intensity in BSA/treated cells. (**b**) Representative dot plots with mean fluorescence intensities (MIF). (**c**) The amounts of thiols expressed as percent of control with BSA (100%). (**a**,**c**) means ± SEM from 6 experiments (biological replicates); *P < 0.05, **P < 0.001, PGLYRP4, KCN, diamide, or CCCP *vs* BSA; *NS* not significant (*P* > 0.05); ^#^*P* < 0.05, ^##^*P* < 0.001 as indicated by brackets.

We next considered the possibility that PGLYRP4-induced membrane depolarization^9,26^ could affect the function of *B. subtilis* and *E. coli* respiratory electron transport chains and in this way either cause or contribute to the PGLYRP4-induced thiol depletion. Treatment of *B. subtilis* with PGLYRP4 for 30 min (the time of maximum PGLYRP4-induced thiol depletion^10^) resulted in significant, but low-level membrane depolarization (1.75-fold change relative to BSA control), similar to membrane depolarization induced by 100 µM KCN, a cytochrome *aa*_3_-600 inhibitor^36–38^ (2.27-fold change), and diamide (2.18-fold change), as measured by a decrease in red fluorescence of membrane potential sensitive probe DiOC_2_(3) (Fig. 9a,b). KCN-induced membrane depolarization is consistent with inhibition of cytochrome *aa*_3_-600 and its proton-pumping ability (BsubCyc https://bsubcyc.org/)^24,36–38^. CCCP, which dissipates both membrane potential and proton gradient, induced strong membrane depolarization as expected (15.2- and 4.9-fold change for 20 µM and 1 µM, respectively), significantly greater than PGLYRP4, KCN, and diamide (Fig. 9a,b).

We next tested whether membrane depolarization with CCCP in *B. subtilis* would induce thiol depletion and whether the extent of thiol depletion with PGLYRP4, diamide, and CCCP would correlate with the extent of membrane depolarization. Both PGLYRP4 and diamide induced severe and highly significant thiol depletion (93–96%) but very low membrane depolarization, whereas CCCP induced significant, but low-level thiol depletion (33% to 40%), significantly less than PGLYRP4- and diamide-induced thiol depletion (Fig. 9c).

**Figure 10:**
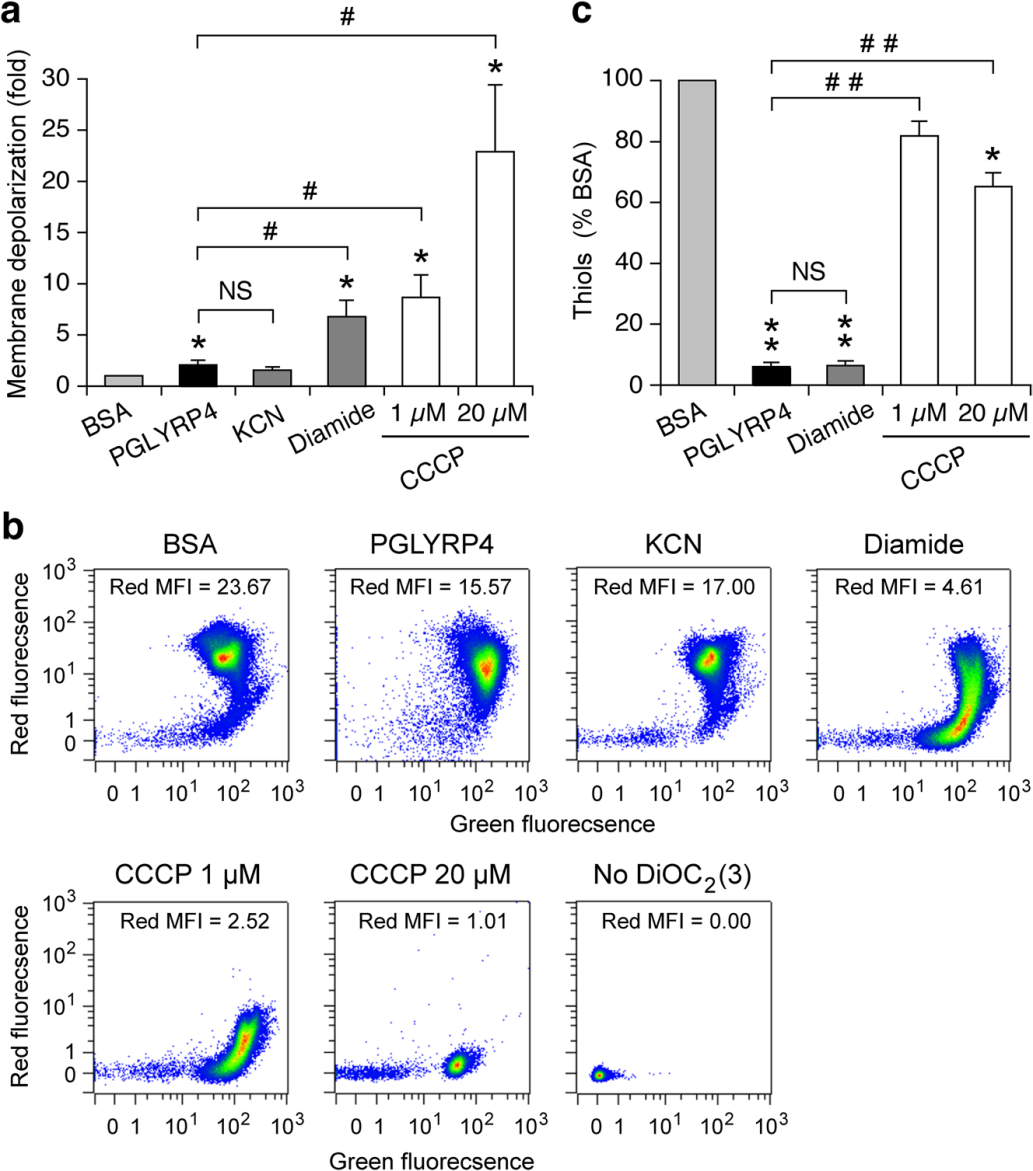
Comparison of membrane depolarization and thiol depletion in *E. coli*. *E. coli* MG1655 were treated with BSA, PGLYRP4, KCN, diamide, or CCCP as indicated for 30 min. (**a**) Membrane depolarization was measured by flow cytometry with membrane potential sensitive dye DiOC_2_(3) and the results are the ratios of mean red fluorescence intensity in BSA/treated cells. (**b**) Representative dot plots with mean fluorescence intensities (MIF). (**c**) The amounts of thiols expressed as percent of control with BSA (100%). Means ± SEM from 6 (**a**) or 4 (**c**) experiments (biological replicates); *P < 0.05, **P < 0.001, PGLYRP4, KCN, diamide, or CCCP *vs* BSA; *NS* not significant (*P* > 0.05); ^#^*P* < 0.05, ^##^*P* < 0.001 as indicated by brackets.

We next tested the relationship between membrane depolarization and thiol depletion in *E. coli*. Similar to the results in *B. subtilis*, in *E. coli* PGLYRP4 induced significant, but very low-level membrane depolarization (2.1-fold change), in contrast to CCCP, which induced high-level membrane depolarization (22.9- and 8.7-fold change for 20 µM and 1 µM, respectively), significantly higher than PGLYRP4 (Fig. 10a,b). 100 µM KCN did not induce significant membrane depolarization, but diamide induced significant membrane depolarization (6.8-fold change), higher than PGLYRP4 and KCN, but lower than CCCP. Also similar to the results in *B. subtilis*, in *E. coli* PGLYRP4 and diamide induced severe thiol depletion (94%), whereas CCCP induced low-level thiol depletion (18–35%, significant for 20 µM CCCP compared with BSA control), but significantly less than PGLYRP4- and diamide-induced thiol depletion (Fig. 10c).

These results both in *B. subtilis* and *E. coli* reveal that there is no correlation between the extent of membrane depolarization and thiol depletion. PGLYRP4 and diamide induced very strong thiol depletion, PGLYRP4 induced low-level membrane depolarization, diamide induced low to medium-level membrane depolarization, and CCCP induced strong membrane depolarization and low-level thiol depletion.

## Discussion

Mammalian PGRPs induce exaggerated oxidative, thiol, and metal stress responses in bacteria that become lethal, but exactly how these stress responses are induced, especially in Gram-positive bacteria, has been unknown^9,10^. In this study we show that in *B. subtilis* four main components of its respiratory electron transport chain, NDH, MK, DMK, and cytochrome *aa*_*3*_-600, are required for efficient PGLYRP4-induced thiol depletion (thiol stress) and bacterial killing. We further show that in *E. coli* similar main components of the respiratory electron transport chain, FDH-O, NDH-1, UQ, and cytochrome *bd*-I, are also required for efficient PGLYRP4-induced thiol depletion (thiol stress). These results are consistent with the requirement for FDH-O, NDH-1, UQ, and cytochrome *bd*-I for efficient PGLYRP4-induced oxidative stress and killing in *E. coli*^11,26^.

**Figure 11:**
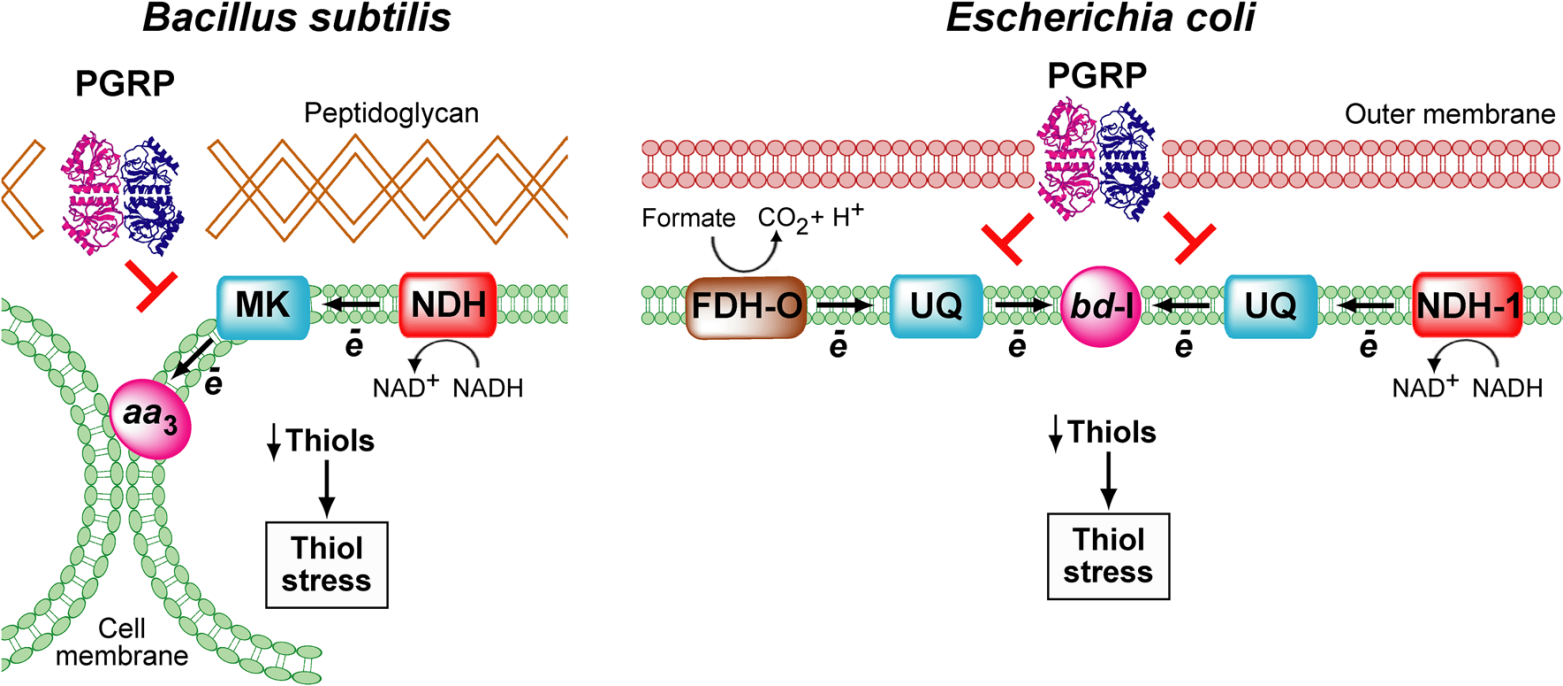
Proposed events in PGRP-induced thiol stress in *B. subtilis* and *E. coli*. PGRP binds to peptidoglycan in the cell separation site in dividing *B. subtilis* or to the outer membrane in *E. coli*^9^ and interferes with the function of the respiratory electron transport chain, consisting of NDH, MK (and DMK), and cytochrome *aa*_*3*_-600 in *B. subtilis*, or FDH-O, NDH-1, UQ, and cytochrome *bd-*I in *E. coli*^11,26^, leading to depletion (oxidation) of cellular thiols and thiol stress.

In the respiratory electron transport chain in both *B. subtilis* and *E. coli* oxidized quinones (MK and DMK in *B. subtilis* and UQ in *E. coli*) accept electrons from NADH (catalyzed by NDH) and also from formate in *E. coli* (catalyzed by FDH-O) and become reduced to quinols. Quinols then serve as electron donors for cytochrome *aa*_*3*_-600 in *B. subtilis* and cytochrome *bd*-I in *E. coli* and become oxidized, and then are reduced again by the dehydrogenases (Fig. 11). Our results show that in both *B. subtilis* (Fig. 6) and in *E. coli*^11^ PGLYRP4 induces a malfunction of the respiratory electron transport chain, which results in a decrease and subsequent shutdown of respiration and could lead to depletion of cellular thiols due to an increase in electrophilic oxidized quinone pool (MK and DMK in *B. subtilis* and UQ in *E. coli*). These oxidized quinones could then directly or indirectly abstract electrons from cellular thiols (Fig. 11). This possibility is supported by our previous results showing an increase in the ratio of oxidized to reduced quinones in PGLYRP4-treated bacteria^26^ and also by attenuation of PGLYRP4-induced thiol depletion in *B. subtilis* Δ*ndh* and Δ*qoxA* and *E. coli* Δ*fdhD*, Δ*fdhE*, Δ*fdoG*, Δ*nuo*, and Δ*cydB* mutants. This attenuation in the Δ*qoxA* and Δ*cydB* deletion mutants could be explained by an increase in the reduced MK and DMK or UQ in these mutants due to the lack of their physiologic electron acceptors (cytochrome *aa*_*3*_-600 and cytochrome *bd*-I), and in the Δ*ndh*, Δ*nuo*, Δ*fdhD*, Δ*fdhE*, and Δ*fdoG* deletion mutants due to a buildup of NADH or formate and a shift in the intracellular redox to a more reduced environment. But other effects of PGLYRP4 disturbing the balance of the respiratory electron transport chain are also possible.

How can PGRPs affect the electron transport chain? In *B. subtilis* (and also other Gram-positive bacteria) PGRPs bind to the separation sites of the newly formed daughter cells, created by bacterial peptidoglycan-lytic endopeptidases, LytE and LytF, which separate the daughter cells after cell division^9^. These cell-separating endopeptidases likely expose PGRP-binding muramyl peptides, as shown by co-localization of PGRP and LytE and LytF at the cell-separation sites, and no binding of PGRP to other regions of the cell wall with highly cross-linked peptidoglycan^9^. This localization is necessary for the bacterial killing, because mutants that lack LytE and LytF endopeptidases and do not separate after cell division do not bind PGRP, and are also not readily killed by PGRP^9^. Binding of PGRP to peptidoglycan likely induces formation of oligomeric PGRP structures^1,39^, which at this cell separation site could possibly gain access to the cell membrane. In Gram-negative bacteria, PGRPs bind to the outer membrane^9^ through a binding site specific for LPS^5,40^, which is outside the peptidoglycan-binding pocket. Binding of PGRP to the outer membrane is required for killing, because exogenous LPS blocks both PGRP binding and PGRP-induced killing of *E. coli*^9^. In both Gram-positive and Gram-negative bacteria PGRPs stay bound to the cell envelope and do not enter the cytoplasm^9^ and, thus, induce oxidative, thiol, and metal stress and bacterial death from this extracellular site. However, it is not known whether PGRPs can directly contact the cell membrane or its components, and thus it is not known whether the effects of PGRPs on the cytoplasmic membrane and its respiratory chain are direct or indirect.

We considered that PGRP-induced membrane damage or depolarization could be the cause of the respiratory chain malfunction and subsequent thiol depletion. However, PGRPs do not induce permeabilization of the cytoplasmic membrane in *B. subtilis* for over 2 h^4^ and in *E. coli* and other bacteria for over 6 h^4,6,9^, whereas maximum thiol depletion is reached in 30 min^10^ and significant killing is accomplished in 1–2 h^4,9,10^. Also, at the time of maximum thiol depletion, PGLYRP4 induced only very low-level membrane depolarization (Figs. 9, 10). Furthermore, there was no correlation between the extent of membrane depolarization and the extent of thiol depletion, i.e., PGLYRP4 induced very low membrane depolarization and almost complete thiol depletion, whereas CCCP induced high membrane depolarization and very low thiol depletion (Figs. 9, 10). Moreover, low concentration of CCCP that caused low level of membrane depolarization had the opposite effect to PGLYRP4 on cellular respiration (Fig. 6), further supporting the notion that CCCP and PGLYRP4 have different effects on the cell membrane and the respiratory electron transport chain.

Our current results are consistent with our previous analysis of gene expression, which showed high induction of thiol stress response genes in both *B. subtilis* and *E. coli* by PGLYRP4^10^, similar to diamide^12,16^, such as chaperones and protein quality control genes. They included several genes activated by CtsR (master regulator of protein quality control and thiol stress response genes) and CssRS (two-component system that detects and disposes of misfolded proteins) in *B. subtilis*, and *ibpAB*, *htpG*, *htpX*, *hspQ*, and *degP* in *E. coli*, indicating high thiol depletion and thiol stress^10^. However, there was no or low increase in the expression of these genes in CCCP-treated *B. subtilis* and *E. coli*^10^, consistent with very limited thiol depletion in response to CCCP (Figs. 9, 10). Altogether, these results suggest that PGLYRP4-induced membrane depolarization may be the consequence of the decreased function of the respiratory electron transport chain rather than the primary cause of its malfunction and thiol depletion. However, other possibilities cannot be excluded, such as membrane depolarization as the initial event, or other effects of PGLYRP4 on the cytoplasmic membrane that interfere with the respiratory chain. Severe down-regulation of the expression of genes for high energy-requiring processes, such as synthesis of flagella and motility in PGLYRP4-treated *B. subtilis* and *E. coli*^10^, are also consistent with the malfunction of the energy-generating respiratory electron transport chain.

In conclusion, our results indicate that in *B. subtilis* and *E. coli* the respiratory electron transport chain components (NDH, MK, DMK, and cytochrome *aa*_*3*_-600 in *B. subtilis*, and FDH-O, NDH-1, UQ, and cytochrome *bd*-I in *E. coli*) are required for efficient PGLYRP4-induced killing and thiol depletion. These results resemble the requirement for similar components of the respiratory electron transport chain in PGLYRP4-induced oxidative stress in *E. coli*^11,26^. Thus, our results indicate conservation of the basic PGRP killing mechanisms in both Gram-positive and Gram-negative bacteria. Targeting these components may aid in the future development of new approaches to reduce bacterial virulence, enhance host resistance to infections, and increase effectiveness of antibacterial therapy.

## Materials and methods

### Materials

Human recombinant PGLYRP4 (used as a representative bactericidal PGRP) was expressed in S2 cells and purified as previously described^4,6^ in a buffer containing 10 mM TRIS (pH 7.6), with 150 mM NaCl, 10 µM ZnSO_4_, and 10% glycerol, and used at 100 µg/ml final concentration (0.87 µM, as PGLYRP4 is a 115 kDa disulfide-linked dimer^4^), unless otherwise indicated. All reagents were from Sigma-Aldrich (unless otherwise indicated), used at the following final concentrations: DPA (diphenylamine, 75 µM), menaquinone-4 (MK_4_, vitamin K2, 40 µg/ml), menaquinone-7 (MK_7_, 40 µg/ml), CCCP (carbonyl cyanide 3-chlorophenylhydrazone from Molecular Probes, 1 µM and 20 µM), diamide (tetramethyldiazenedicarboxamide, 250 µM), fatty acids-free purified bovine serum albumin (BSA, 100 µg/ml used as a negative control). For stock solutions made in DMSO, equivalent amounts of DMSO were added to all groups.

### Bacteria, growth, and media

*B. subtilis* and *E. coli* strains used in this study are listed in Supplementary Tables S3 and S4. Bacteria were grown aerobically overnight at 37 °C in an orbital shaker (250 rpm) in 5 ml LB in a 50-ml tube, diluted to OD_660_ = 0.01 into fresh 5 ml LB in a 50-ml tube and grown to OD_660_ = 0.6. Bacteria were centrifuged and suspended in the “Assay Medium”, which for *B. subtilis* was: TRIS-Schaeffer medium with 0.05% NH_4_Cl, 5 µM ZnSO_4_, 5% glycerol, 0.2% glucose, with addition of 2% of 100% LB, and for *E. coli* was: 5 mM TRIS (pH 7.6) with 2.5% glycerol, 150 mM NaCl, 5 µM ZnSO_4_, and 1% of 100% LB (final concentrations). These assay media were the same for all current and previous experiments on *B. subtilis* and *E. coli*^4,9–11,26^.

### Screening of *B. subtilis* Tn-mutant library

Highly saturated Tn insertion library in *B. subtilis* 168 containing ~ 138,000 unique mutants, generated using *magellan6x* and characterized previously^19,20^, was used in this study. Each mutant contained one transposon randomly inserted into the chromosome and the library contained insertions in 63% of all TA sites and ~ 26 different Tn mutants for each gene/intergenic region, with nearly random distribution of insertions and the absence of large gaps or hot spots^19,20^. For each experiment, 5 µl of freshly thawed glycerol stock of the Tn library was inoculated into 5 ml of LB and grown aerobically at 37 °C in an orbital shaker (250 rpm) for 2 generations. The bacteria were sedimented by centrifugation and suspended at ~ 2.5 × 10^6^ bacteria/ml in 50 µl of fresh warm Assay Medium with addition of 100 µg/ml PGLYRP4, or 150 µg/ml PGLYRP4, or 150 µg/ml BSA (control) and incubated at 37 °C aerobically with 250 rpm shaking for 3 h. These PGLYRP4 treatments resulted in ~ 83% and ~ 99.5% reduction in CFU, respectively (higher concentration of PGLYRP4 than in other assays was required to achieve ~ 99.5% reduction in CFU because of the high number of bacteria used in these experiments); BSA treatment resulted in ~ 20-fold increase in CFU in 3 h, compared with the initial inoculum. After 3-h incubation each entire 50 µl culture was diluted with TRIS-Schaeffer medium and plated on LB agar for single colonies on multiple 15 cm plates, which were then incubated at 37 °C aerobically overnight. Approximately 100,000, 8,000, and 230 colonies from BSA- and PGLYRP4-treated groups, respectively, were then scraped from the plates and thoroughly mixed. For each treatment group, genomic DNA was extracted from 1 ml aliquot of pooled colonies containing ~ 5 × 10^9^ CFU using a Gentra Puregene Yeast/Bacteria Kit (Qiagen). The entire experiment was repeated 3 times (3 biological replicates).

### Preparation of DNA of *B. subtilis* Tn insertions

Extracted genomic DNA from pooled colonies was precipitated using sodium acetate (pH 5.2) and ethanol and dissolved in 50 μl of 10 mM TRIS–HCl buffer (pH 8.5). 6 μg of the genomic DNA was then digested for 3 h at 37 °C with 3 μl of Mmel (2,000 unites/ml), 0.66 μl of 32 μM S-adenosylmethionine, 30 μl of New England Biolabs (NEB) buffer 4 and H_2_O added to 300 μl total volume. The reaction was stopped by incubation with 6 units of calf intestinal phosphatase (NEB) at 37 °C for 60 min. The digested genomic DNA fractions were recovered in 300 μl phenol:chloroform:isoamyl alcohol (25:24:1) and precipitated with sodium acetate and ethanol. The DNA was dissolved in 27.5 μl of 2 mM TRIS–HCl buffer (pH 8.5) for 2 h at 37 °C and subjected to deep sequencing analysis.

### Transposon sequencing (Tn-seq)

Illumina sequencing was carried out as described previously^19,20,26^. Briefly, we prepared the DNA adaptor for Illumina by annealing oligonucleotides oCJ25 and oCJ26 (Supplementary Table S5). The oligonucleotides were mixed in equal volumes and placed at 96 °C for 2 min; 2 μl of annealed adaptor was ligated to processed genomic DNA using 1.5 μl of T4 DNA ligase and 3.5 μl of 10 × ligase buffer from NEB at 16 °C overnight and then purified with Qiagen PCR purification kit and eluted with 50 μl of 10 mM TRIS–HCl buffer (pH 8.5). 2 μl of each DNA mixture was used as a template in a 50 μl PCR reaction for 20 cycles using primers oCJ23 and oCJ22 (Supplementary Table S5). Amplified DNA was purified using 1% agarose gel electrophoresis, and the 128 bp band was recovered in 14 μl 10 mM TRIS–HCl buffer (pH 8.5) with Qiagen DNA Gel-extraction mini kit. The sequencing of the Tn-chromosome junctions was performed using a MiSeq 2000 (Illumina) at the Tufts University Core Facility, Boston, MA with oCJ24 and oCJ27 primers to obtain unique barcode sequences for each experimental group (Supplementary Table S5). The complete Tn-seq results have been deposited in NCBI SRA (https://www.ncbi.nlm.nih.gov/sra) with the accession number PRJNA628733 (BioSamples SAMN14749977-SAMN14749985).

### Tn-seq data analysis

Read mapping and calculations were carried out on the Tufts University Galaxy server as described previously^19,20,26,41^. Briefly, Illumina reads were aligned to the *B. subtilis* 168 genome sequence using Bowtie^42^. The Tufts Galaxy server custom script was applied to enumerate read number for each insertion site. The insertion sites were then aggregated by annotated genes. In order to fully cover all genes and promoter regions, a GenBank file containing all intergenic regions was generated to allow insertion sites to aggregate to intergenic regions. The predicted number of reads for each gene was calculated based on the length of the gene and the total number of reads in the library. We identified 4142 genes and 1824 intergenic regions that contained 3 or more Tn insertion sites and we used these genes for further analysis, because ≥ 3 insertion sites are needed for reliable identification of mutants with changed frequency in the library^19,20,26,41^. For each gene in both BSA- and PGLYRP4-treated groups we calculated Dval, which represents the predicted frequency of each gene in the library based on the length of the gene, the total length of the genome, and the total number of reads in the library; Dval = the number of actual reads for the gene/predicted number of reads for the gene. For each gene we then calculated the survival index, SI = Dval in PGLYRP4-treated group/Dval in control BSA-treated group. The SI reflects a change in the frequency of each gene (Tn insertion mutant) following PGLYRP4 treatment. A neutral mutation with no effect on the survival in the presence of PGLYRP4 has SI = 1, whereas SI > 1 denotes a mutation that makes bacteria more resistant to PGLYRP4, and SI < 1 denotes a mutation that makes bacteria more sensitive to PGLYRP4.

### Construction and verification of deletion mutants

We created *B. subtilis* gene deletion mutants (Supplementary Table S3) by replacing the entire coding sequence of the genes (in frame) with kanamycin resistance cassette using homologous recombination^27^. Briefly, we amplified ~ 1 kb of the 5′ and 3′ flanking sequences of the target gene using the 5pL/5pR and 3pL/3pR primers and the kanamycin resistance cassette using the Kan-F and Kan-R primers (Supplementary Table S5). The amplified kanamycin resistance cassette and the gene specific flanking sequences were purified, joined, and re-amplified using the 5pL and 3pR primers, and transformed into wild type *B. subtilis* using the natural competence protocol^27^. Briefly, 0.5 ml of competent cells was mixed with the gene targeting PCR product and incubated at 37 °C for 30 min with shaking (150 rpm). 200 μl of transformed cells were plated on LB agar with 10 μg/ml kanamycin and after 24-h incubation at 37 °C kanamycin resistant colonies were picked and streaked on new LB agar plates with 10 μg/ml kanamycin. The gene deletions were verified by colony PCR with kanamycin-specific Kan-2 F primer and gene-specific 3′ flanking sequence 3pR primers (Supplementary Table S5). We have also performed second validation by PCR using primer pairs specific for the coding sequence of the deleted gene (Supplementary Table S5). Gene deletion was confirmed by the presence of ~ 1.2 kb product from PCR on the parental wild-type strain and the absence of this product from PCR on the deletion mutant. Construction of *E. coli* deletion mutants (Supplementary Table S4) was previously described^26,43^.

### PGLYRP4 killing assay

*B. subtilis* were grown as described above in “Bacteria, growth, and media” section and suspended at 0.25 × 10^6^ bacteria/ml in 50 µl of fresh warm Assay Medium with addition of 100 µg/ml BSA or PGLYRP4 and incubated at 37 °C aerobically with 250 rpm shaking for 3 h. The numbers of viable bacteria (CFU) were then determined by colony counts^9,10^. To determine the role of MK in PGLYRP4 killing, 75 µM of DPA was used as MK synthesis inhibitor^28,29^ for the entire 3-h duration of the killing experiment on *B. subtilis* 6633. To reverse the effects of mutations on MK synthesis, 40 µg/ml of MK_4_ was added to the mutants and the parental strain both during the growth in LB of the diluted cultures in the morning and to the Assay Medium during the 3-h killing experiment. To reverse the effects of DPA on MK synthesis, 40 µg/ml of MK_4_ was added to the Assay Medium (with or without DPA) during the 3-h killing experiment.

### Menaquinone assay

Menaquinone (MK) was extracted as previously described^26,44,45^. *B. subtilis* 6633 and its Δ*glpD* and Δ*aroB* deletion mutants were grown overnight in LB as described above in “Bacteria, growth, and media” section and then suspended at OD_660_ = 0.05 in 2.5 ml of fresh warm Assay Medium and incubated in 40-ml glass tubes at 37 °C aerobically with 250 rpm shaking until the cultures reached OD_660_ = 0.3, when 7.5 ml of ice-cold methanol followed by 7.5 ml of petroleum ether at 40–60 °C were added. The samples were vigorously vortexed for 5 min and centrifuged for 2 min at 900×*g* at 4 °C, and the upper petroleum ether phase was transferred to a glass test tube and the remaining cell suspension was re-extracted with another 7.5 ml of petroleum ether, which was combined with the first extract and evaporated to dryness. The samples were reconstituted in 50 µl of 100% methanol just prior to the analysis, and MK_7_ was quantified by LC/MS/MS as previously described^26,46^ at the Purdue University Bindley Bioscience Center Core Facility, West Lafayette, IN. An Agilent 6460 QQQ coupled to an Agilent 1200 Rapid Res LC system with Water’s Xterra C18 2.1 × 100 mm, 3.5 µm column was used for the LC separation as previously described^26^. The extraction and evaporation in the air converted all MK to an oxidized form and only oxidized MK_7_ and no reduced form (< 0.3 ng/culture) was detected. MK_7_ (from Cayman Chemicals) was used as a standard and Agilent Masshunter Quantitative analysis (v6.0) was used for the data analysis^26^. The results are shown as total ng MK_7_/culture.

### Thiols assay

Depletion (oxidation) of thiols (all reduced sulfhydryl groups) was measured as previously described^10^. *B. subtilis* and *E. coli* were grown and suspended in 50 µl of Assay Medium at OD_660_ = 0.15 as described above in “Bacteria, growth, and media” section, with addition of 100 µg/ml BSA (negative control), 250 µM diamide (positive control), or 100 µg/ml PGLYRP4, or 1 µM or 20 µM CCCP. Bacteria were incubated aerobically at 37 °C on a shaker for 30 min. To determine the role of MK in PGLYRP4-induced thiol depletion, 75 µM of DPA (MK synthesis inhibitor^28,29^) was added both during the growth in LB of the diluted *B. subtilis* 6633 cultures in the morning and to the Assay Medium during the 30-min thiol depletion incubation. To reverse the effects of mutations or DPA on MK synthesis, 40 µg/ml of MK_7_ was added to the mutants and the parental strain both during the growth in LB of the diluted cultures in the morning and to the Assay Medium during the 30-min thiol depletion incubation. The reactions were then stopped by lysing bacteria with 40 mM HEPES, 4 mM EDTA, 400 mM KCl, 0.2% Triton X-100, pH 8.1 in a bath sonicator on ice for 5 min, and total amounts of reduced thiols were determined using fluorescent Measure-iT Thiol Assay Kit (Invitrogen/Molecular Probes) according to the manufacturer’s instructions. Total concentration of thiols was calculated as µM from a standard curve using glutathione and reported as % of BSA-containing cultures. CCCP caused 10% to 30% interference with the readout of the thiol assay and for calculating the results of thiol depletion by CCCP standard curves containing 1 µM or 20 µM CCCP were used. 100 µM KCN highly interfered with the thiol assay and for this reason thiol depletion in KCN-treated bacteria could not be reliably determined.

### Oxygen consumption and extracellular acidification

O_2_ consumption rate (OCR, which measures total aerobic respiration) and extracellular acidification rate (ECAR, which measures total H^+^ production) were assayed simultaneously in real time with the Seahorse XFp analyzer (Agilent Technologies), which uses label-free, solid-state sensor cartridges in a microplate format^11,33^. First, to attach *B. subtilis* to the XFp cell culture microplates, the wells were pre-coated for 20 min at room temperature with 25 µl/well of BD Cell-Tak Cell and Tissue Adhesive (BD Biosciences, prepared by diluting 46 µl of Cell-Tak into 3 ml of 0.1 M NaHCO_3_ and then neutralizing it with HCl), followed by rinsing the wells twice with 200 µl of sterile distilled H_2_O and drying at room temperature for 20 min. *B. subtilis* 6633 were grown and diluted to OD_660nm_ = 0.0125 with warm Assay Medium as described in “Bacteria, growth, and media” section, and 40 µl/well of bacteria was dispensed into the Cell-Tak-coated wells (2 × 10^6^ *B. subtilis* per well). *B. subtilis* were then sedimented by centrifugation at 2270×*g* for 2 min, 100 µg/ml BSA (negative control), or 100 µg/ml PGLYRP4, or 1 µM CCCP (positive control) were added and incubation at 37 °C and OCR and ECAR measurements were started immediately. The micro-chamber wells are automatically re-equilibrated and oxygenated between measurements through the up and down mixing by the probes of the XFp sensor cartridge. The results are reported as mean ± SEM OCR (in pmol/min) and ECAR (in mpH/min) for the original bacterial incubation mixture, and also as phenograms of OCR plotted *versus* ECAR that illustrate the total cell energy phenotype.

### Membrane depolarization

Membrane depolarization was measured by flow cytometry with the membrane potential sensitive fluorescent probe DiOC_2_(3) (3,3-diethyloxacarbocyanine iodide) using Bac*Light* Bacterial Membrane Potential Kit (from Molecular Probes, ThermoFisher Scientific B34950)^26,47^ as recommended by the manufacturer. DiOC_2_(3) accumulates in the cell membrane and changes fluorescence from green to red at high membrane potential, and thus membrane depolarization is reflected by a decrease in red fluorescence. *B. subtilis* 168 or *E. coli* MG1655 were grown and suspended in 50 µl of Assay Medium at OD_660_ = 0.02 as described above in “Bacteria, growth, and media” section. DiOC_2_(3) (30 µM) and BSA (100 µg/ml, negative control), PGLYRP4 (100 µg/ml), KCN (100 µM), diamide (250 µM), or CCCP (1 µM or 20 µM, positive control at concentrations that caused partial or complete membrane depolarization) were added, cultures were incubated aerobically at 37 °C on a shaker for 30 min, and fluorescence of ~ 2 × 10^4^ bacteria/culture was immediately measured by flow cytometry using MACSQuant (Miltenyi) flow cytometer with FITC (green) and PE (red) excitation and emission settings for green and red DiOC_2_(3) fluorescence. The extent of membrane depolarization was expressed as the ratios of mean red fluorescence intensity in BSA/treated cells ± SEM, with representative dot plots of green and red fluorescence intensity also shown.

### Gene expression arrays

Preparation of the whole genome expression arrays was described previously^10^ and the entire data for all the arrays were deposited in NCBI GEO with accession number GSE44212. Here, from these arrays, we present and analyze for albumin- and PGLYRP4-treated *B. subtilis* the expression of genes for the synthesis of shikimate and for NDH and cytochromes, which are the subject of this study and were not analyzed or presented previously. Briefly, exponentially growing *B. subtilis* 168 were suspended in the Assay Medium at OD_660_ = 0.1 as in “Bacteria, growth, and media” section, and incubated aerobically for 30 min at 37 °C with 100 µg/ml albumin (control) or 100 µg/ml PGLYRP4 with 250 rpm shaking. Bacteria were disrupted by shaking with Zirconia beads and RNA was then extracted using Ambion RiboPure-bacteria RNA extraction kit, cDNA was synthesized with random hexamer primers, fragmented, labeled with terminal transferase and biotin, and hybridized to whole genome Affymetrix 900513 GeneChip *B. subtilis* Genome Array using Affymetrix Hybridization Oven 640 and Affymetrix GeneChip Fluidics Station 450 and protocols provided by Affymetrix GeneChip Technical Manual. Scanning and data extraction were done using Affymetrix GeneChip Scanner 3000 and protocols provided by Affymetrix GeneChip Technical Manual. cDNA synthesis, labeling, hybridization, and scanning were performed at the Genomic and RNA Profiling Core facility, Baylor College of Medicine, Houston, TX. The entire experiment was repeated 3 times. Hybridization intensity data signals were normalized and analyzed as described^10^ using Affymetrix GeneChip Command Console Software. Signal intensities from 3 experiments (3 biological replicates) were used to calculate geometric means ± SEM. Transformed Ln(signal intensity) values were used for direct statistical comparisons of expression signals between PGLYRP4-treated and control (albumin) groups. The expression of the relevant genes for the synthesis of quinones, FDH, and cytochromes in BSA- and PGLYRP4-treated *E. coli* was reported previously^26^.

### Annotation of gene functions

The functions of *B. subtilis* and *E. coli* genes were annotated using the BsubCyc (https://bsubcyc.org/)^24^ and EcoCyc (https://ecocyc.org/)^48^ databases.

### Statistical analyses

Quantitative results are presented as arithmetic or geometric means ± SEM, with statistical significance of the differences between groups determined by the two-sample two-tailed Student’s *t*-test or paired Student’s *t*-test using Microsoft Excel; *P* ≤ 0.05 was considered significant. The *n* and *P* values are indicated in the figures.

## Supplementary Information

Supplementary Information.

## Data Availability

All data generated or analyzed during this study are included in this published article (and its Supplementary Information) or have been deposited in NCBI as cited in the text under the accession numbers PRJNA628733 and GSE44212.
