# Supplementary material for: Respiratory chain components are required for peptidoglycan recognition protein-induced thiol depletion and killing in Bacillus subtilis and Escherichia coli
Source: Sci Rep. 2021 Jan 8;11:64. doi: 10.1038/s41598-020-79811-z (PMC7794252; doi:10.1038/s41598-020-79811-z)
Supplement: Supplementary file 1 — Supplementary Information. [file 41598_2020_79811_MOESM1_ESM.pdf]

Supplementary Information

Respiratory chain components are required for peptidoglycan recognition protein-induced thiol depletion and killing in *Bacillus subtilis* and *Escherichia coli*

Chun-Kai Yang, Des R. Kashyap, Dominik A. Kowalczyk, David Z. Rudner, Xindan Wang, Dipika Gupta, Roman Dziarski

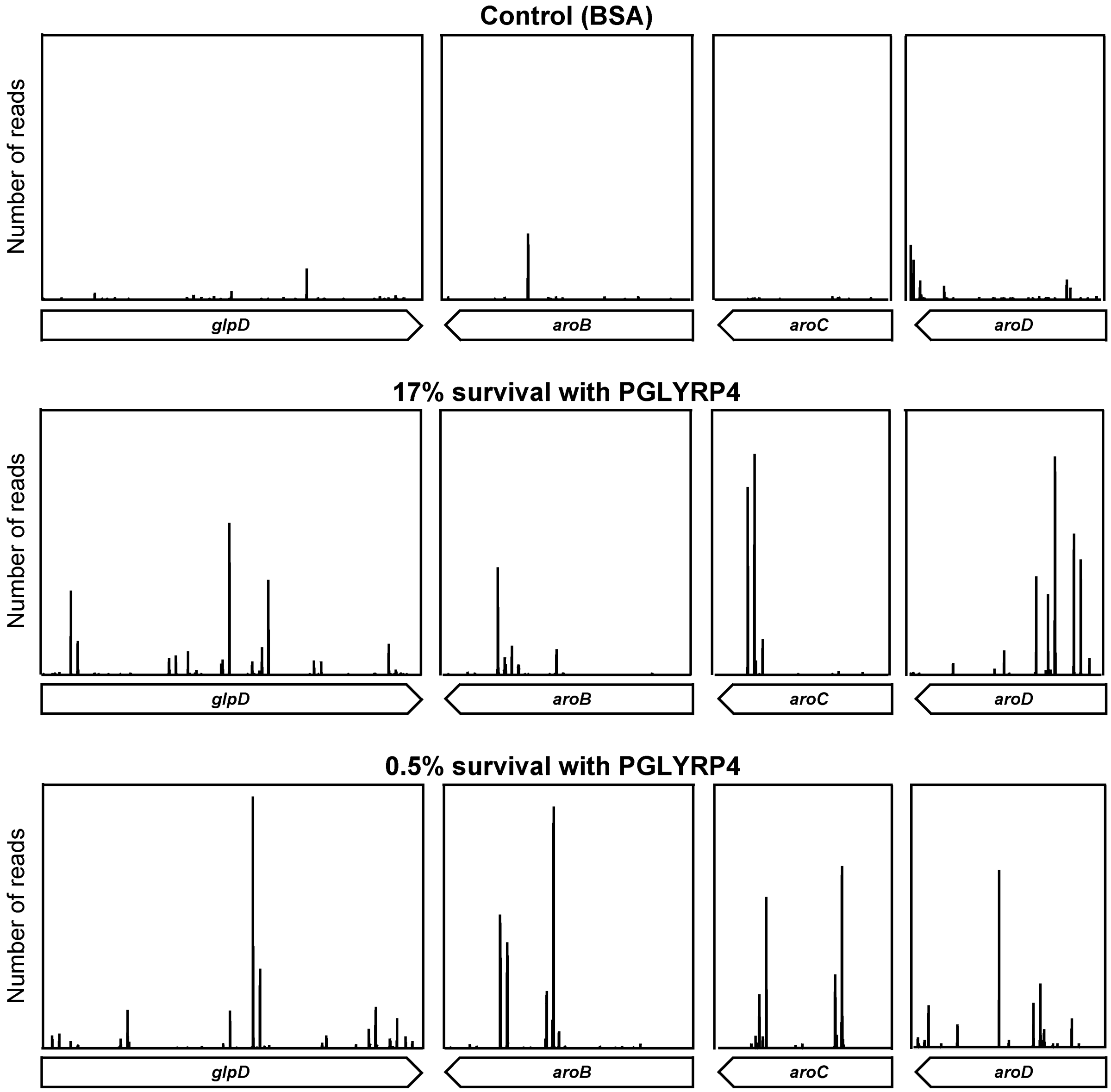

Supplementary Figure S1. Tn insertion sites and the numbers of reads for shikimate synthesis genes from Fig. 1 with high survival index in PGLYRP4-treated cultures. *B. subtilis* Tn insertion library was treated for 3 hrs with BSA as a control or with PGLYRP4 at bactericidal (0.5% survival) or sub-bactericidal (17% survival) concentration, and the numbers of reads for each Tn-insertion site in each gene was determined by Tn-seq. The height of each vertical line represents geometric mean of the number of sequencing reads at this position from 3 independent experiments (biological replicates). The complete Tn-seq results have been deposited in NCBI SRA with accession number PRJNA628733.

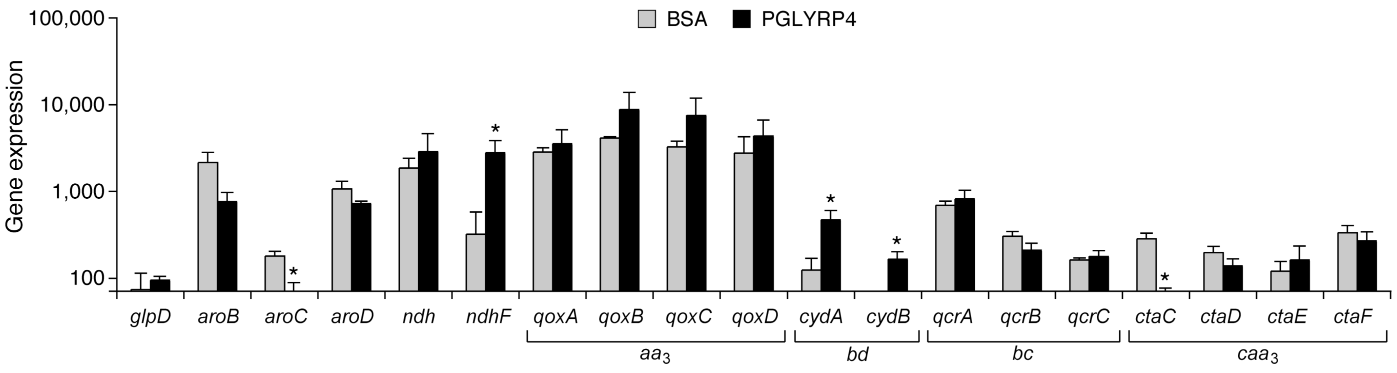

Supplementary Figure S2. Expression of genes in PGLYRP4-treated *B. subtilis* studied in this paper. *B. subtilis* 168 was treated with BSA or PGLYRP4 for 30 min and expression of the indicated genes was determined by whole genome expression arrays (NCBI GEO GSE44212). The results are geometric means ± SEM of signal intensities from 3 independent experiments (biological replicates); Y axis starts at the detection limit; * $P < 0.05$, PGLYRP4 *vs* BSA.

Supplementary Table S1. ***B. subtilis*** **Tn-seq mutants with significantly increased frequency in the Tn-seq library treated with bactericidal concentration of PGLYRP4.[a]**

Gene symbol	Protein	LOG2 SI	Fold *vs* BSA	*P* vs BSA
smiA (yvzG)	conserved protein of unknown function	4.68	25.65	0.0460
aroB	**3-dehydroquinate synthase**	4.64	24.91	0.0186
aroC	**3-dehydroquinate dehydratase**	4.62	24.53	0.0055
mntR	transcriptional regulator (H_2O_2 sensing, regulated by Mn^{2+})	4.53	23.05	0.0109
xtrA	phage PBSX; conserved protein of unknown function	4.34	20.24	0.0048
sacT	transcriptional antiterminator of sacAP expression	3.81	14.05	0.0302
glpD	**sn-glycerol-3-phosphate dehydrogenase**	3.80	13.96	0.0027
adhB	zinc-type alcohol dehydrogenase	3.67	12.74	0.0098
yrkQ	two-component sensor histidine kinase (YrkP)	3.66	12.68	0.0102
efeM	lipoprotein binding ferrous or ferric iron for transport	3.51	11.39	0.0342
clpQ	two-component ATP-dependent protease	3.45	10.91	0.0490
glpP	**sn-glycerol-3-phosphate responding transcription antiterminator (activator of *glpD* transcription)**	3.41	10.61	0.0457
yrdF	putative ribonuclease inhibitor	3.19	9.10	0.0304
moeB	molybdopterin biosynthesis adenylyltransferase	3.10	8.60	0.0000
ccpN	negative regulator of gluconeogenesis (transcriptional repressor)	3.10	8.60	0.0072
yoqT	conserved hypothetical protein	2.95	7.71	0.0374
menE	O-succinylbenzoyl-CoA synthetase	2.81	7.00	0.0491
ksgA	dimethyladenosine 16S ribosomal RNA transferase	2.45	5.46	0.0026
tatCY	component of the twin-arginine pre-protein translocation pathway	2.34	5.06	0.0450
ypmT	conserved hypothetical protein	2.26	4.78	0.0019
lysP	lysine permease	2.24	4.72	0.0452
yqzI	hypothetical protein	2.06	4.18	0.0055
yrhP	putative amino acid exporter	2.06	4.16	0.0519
pyrE	orotate phosphoribosyltransferase	1.99	3.98	0.0143
atpG	ATP synthase (subunit gamma, component F1)	1.99	3.98	0.0446
ylqC	putative RNA binding protein	1.95	3.87	0.0413
yhjQ	copper storage protein Csp3	1.85	3.61	0.0201
relA	GTP pyrophosphokinase (RelA/SpoT)	1.70	3.25	0.0089
yqjK	ribonuclease Z	1.68	3.21	0.0000
gmuC	oligo-alpha-mannoside phosphotransferase system enzyme IIC	1.65	3.15	0.0132
ybyB	conserved protein of unknown function	1.64	3.12	0.0469
bcrC	undecaprenyl pyrophosphate phosphatase	1.59	3.02	0.0001
ykvT	cell wall hydrolase related to spore cortex-lytic enzymes	1.53	2.88	0.0238
dnmA	DNA methyltransferase	1.52	2.87	0.0334
gbsR	transcriptional repressor for gbsAB	1.51	2.85	0.0016
ansR	transcriptional regulator of ansAB (Xre family)	1.45	2.73	0.0461
glnA	glutamine synthetase	1.42	2.68	0.0000
iscS	cysteine desulfurase	1.40	2.64	0.0465
ywqB	putative replication initiation protein	1.37	2.58	0.0065
ywbC	glyoxalase I; hemithioacetal-bacillithiol lactoyl-bacillithiol formation	1.14	2.21	0.0016
yabP	spore protein involved in the shaping of the spore coat	1.14	2.21	0.0404
rplE	ribosomal protein L5 (BL6)	1.11	2.16	0.0019

ywfM	putative toxic excess metabolite exporter	1.07	2.10	0.0290
ubiX	phenolic acid decarboxylase-flavin prenyltransferase subunit	1.00	1.99	0.0539
odhB	2-oxoglutarate dehydrogenase complex	0.95	1.93	0.0017
yycE	conserved hypothetical protein	0.94	1.91	0.0234
liaH	modulator of liaIHGFSR (yvqIHGFEC) operon expression	0.89	1.86	0.0027
ybgB	conserved protein of unknown function	0.88	1.85	0.0208
ydjA	ype-2 restriction enzyme BsuMI component BsuRC	0.76	1.69	0.0026
pksA	transcriptional regulator of bacillaene synthesis operon	0.58	1.50	0.0318

[a] *B. subtilis* Tn insertion library was treated for 3 hrs with PGLYRP4 at bactericidal concentration (150 µg/ml, 0.5% survival) or with BSA as a control, and the survival index (SI) with PGLYRP4, relative to BSA, for individual Tn-mutants was determined by Tn-seq. The results are means from 3 independent experiments (biological replicates) shown as Log2 values of SI and fold increase in Tn mutants' frequency in PGLYRP4-treated cultures compared with BSA-treated cultures (calculated as anti-Log2 of SI). The significance (*P*) of deference in the frequency of Tn mutants in PGLYRP4-treated *versus* BSA-treated cultures was calculated on Log2 SI by Student's *t*-test. The *aroB*, *aroC*, and *glpD* genes studied in this paper, and also *glpP* (activator of *glpD* transcription) are in bold. The volcano plot of all the Tn mutants is shown in Fig. 1. The complete Tn-seq results have been deposited in NCBI SRA with accession number PRJNA628733.

Supplementary Table S2. ***B. subtilis*** **Tn-seq mutants with significantly increased frequency in the Tn-seq library treated with sub-bactericidal concentration of PGLYRP4.**[a]

Gene symbol	Protein	LOG2 SI	Fold *vs* BSA	*P* vs BSA
ycsD	putative hydroxyacyl-[acyl carrier protein] dehydratase	4.40	21.05	0.0011
panB	ketopantoate hydroxymethyltransferase	4.33	20.07	0.0114
racA	chromosome-pole-anchoring protein RacA	4.01	16.16	0.0008
yqaE	transcriptional repressor of yqaF-yqaN operon	3.77	13.62	0.0355
aroC	**3-dehydroquinate dehydratase**	3.77	13.61	0.0265
yvcJ	GTPase, possible regulator of sRNA degradation	3.66	12.67	0.0460
glpP	**sn-glycerol-3-phosphate responding transcription antiterminator (activator of *glpD* transcription)**	3.64	12.49	0.0441
glpD	**sn-glycerol-3-phosphate dehydrogenase**	3.51	11.36	0.0007
purB	adenylosuccinate lyase	3.33	10.09	0.0034
aroB	**3-dehydroquinate synthase**	3.32	9.99	0.0016
gpsA	NADPH-dependent glycerol-3-phosphate dehydrogenase	3.21	9.27	0.0396
yvrG	sensor histidine kinase involved in cell wall processes	3.05	8.31	0.0285
psd	phosphatidylserine decarboxylase proenzyme	3.03	8.15	0.0176
yhcD	hypothetical protein	3.02	8.09	0.0077
ccpA	transcriptional regulator of catabolite repression (Lacl family)	2.86	7.27	0.0358
ygaB	hypothetical protein	2.82	7.06	0.0508
pstBB	phosphate ABC transporter (ATP-binding protein)	2.70	6.49	0.0449
trmK	tRNA (adenine^{22}-N^1)-methyltransferase	2.62	6.16	0.0201
metQ	methionine ABC transporter (ATP-binding protein)	2.49	5.63	0.0014
xtrA	phage-like element PBSX	2.48	5.60	0.0111
yfkO	NAD(P)H-flavin oxidoreductase (nitroreductase)	2.45	5.48	0.0265
yhaX	putative hydrolase	2.42	5.36	0.0167
natK	NatK two-component sensory histidine kinase	2.41	5.31	0.0390
ykzU	hypothetical protein	2.36	5.12	0.0289
opuBD	choline ABC transporter (permease)	2.35	5.12	0.0079
dck	deoxyadenosine/deoxycytidine kinase	2.30	4.93	0.0042
yrkO	putative integral inner membrane protein	2.21	4.61	0.0314
fabZ	3-hydroxyacyl-[acyl-carrier-protein] dehydratase	2.05	4.15	0.0233
yfiU	putative MFS efflux transporter	2.04	4.12	0.0098
yorC	conserved protein of unknown function	2.04	4.10	0.0529
ydhD	spore cortex lytic enzyme	1.99	3.98	0.0017
murP	N-acetylmuramic acid PTS permease-MurP subunit	1.92	3.78	0.0285
atpB	ATP synthase (subunit a, component F0)	1.84	3.59	0.0004
yitS	fatty acid kinase fatty acid binding subunit B	1.83	3.55	0.0000
ydiO	DNA-methyltransferase (cytosine-specific)	1.76	3.38	0.0442
ydcF	conserved protein of unknown function	1.75	3.35	0.0156
pdhB	pyruvate dehydrogenase (E1 beta subunit)	1.74	3.33	0.0130
yckA	amino acid ABC transporter (permease subunit)	1.68	3.20	0.0315
kinD	histidine kinase phosphorylating Spo0A	1.65	3.14	0.0017
yqjI	$NADP^+$-dependent 6-P-gluconate dehydrogenase	1.63	3.09	0.0059
bacE	efflux protein for bacilysin excretion	1.60	3.03	0.0429
ydcI	RNA helicase transcriptional accessory protein	1.60	3.02	0.0542

ybdG	putative hydrolase/transferase	1.53	2.88	0.0122
ykuH	conserved protein of unknown function	1.53	2.88	0.0445
ytxO	outer spore coat protein	1.41	2.66	0.0198
yvbI	conserved protein of unknown function	1.39	2.62	0.0249
gapA	glyceraldehyde-3-phosphate dehydrogenase 1	1.34	2.53	0.0542
yllA	putative cysteine ligase BshC	1.33	2.52	0.0506
pbuG	guanine/hypoxanthine permease	1.32	2.50	0.0025
uvrX	lesion bypass phage DNA polymerase	1.31	2.49	0.0271
serB	phosphoserine phosphatase	1.30	2.46	0.0397
qoxC	cytochrome aa3-600 quinol oxidase (subunit III)	1.27	2.42	0.0225
ycgJ	xenotiotic metabolite methyltransferase	1.25	2.38	0.0446
xkdB	phage PBSX; conserved hypothetical protein	1.22	2.33	0.0180
dgcW	diguanylate cyclase	1.18	2.26	0.0171
asnC	putative DNA-binding transcriptional dual regulator	1.13	2.19	0.0540
ywjA	putative ABC lipid transporter (ATP-binding protein)	1.06	2.08	0.0254
cotC	spore coat protein (outer)	1.03	2.04	0.0452
ftsA	cell-division protein essential for Z-ring assembly	1.02	2.02	0.0270
spoIIIAF	stage III sporulation protein (feeding tube apparatus)	1.01	2.02	0.0067
sacT	transcriptional antiterminator of sacAP expression	0.98	1.97	0.0004
acoR	acetoin dehydrogenase operon transcriptional activator AcoR	0.90	1.86	0.0008
ppsE	nonribosomal plipastatin synthetase E	0.86	1.82	0.0001
ggaA	poly(glucosyl N-acetylgalactosamine 1-phosphate) glucosyltransferase	0.82	1.77	0.0109
sdhC	succinate dehydrogenase cytochrome b558 subunit	0.82	1.77	0.0151
ybaR	putative permease	0.76	1.69	0.0066
xylB	xylulose kinase	0.64	1.56	0.0267
yorL	DNA polymerase with 3'-5' exonuclease activity	0.63	1.55	0.0143
resE	ResE two-component sensory histidine kinase	0.62	1.53	0.0048
yvcB	conserved protein of unknown function	0.50	1.41	0.0003
dhbF	siderophore 2,3-dihydroxybenzoate-glycine-threonine trimeric ester bacillibactin synthetase	0.39	1.31	0.0509

[a] *B. subtilis* Tn insertion library was treated for 3 hrs with PGLYRP4 at sub-bactericidal concentration (100 µg/ml, 17% survival) or with BSA as a control, and the survival index (SI) with PGLYRP4, relative to BSA, for individual Tn-mutants was determined by Tn-seq. The results are means from 3 independent experiments (biological replicates) shown as Log2 values of SI and fold increase in Tn mutants' frequency in PGLYRP4-treated cultures compared with BSA-treated cultures (calculated as anti-Log2 of SI). The significance (*P*) of deference in the frequency of Tn mutants in PGLYRP4-treated *versus* BSA-treated cultures was calculated on Log2 SI by Student's *t*-test. The *aroB*, *aroC*, and *glpD* genes studied in this paper, and also *glpP* (activator of *glpD* transcription) are in bold. The volcano plot of all the Tn mutants is shown in Fig. 1. The complete Tn-seq results have been deposited in NCBI SRA with accession number PRJNA628733.

Supplementary Table S3. *Bacillus subtilis* strains used in this study.

***Bacillus subtilis* strain**	**Relevant genotype**	**Source or reference**
Bacillus subtilis 168	*Bacillus subtilis* subsp. *subtilis*, parent strain for mutants, trpC2	Laboratory stock [9, 10, 27]
Bacillus subtilis 6633	*Bacillus subtilis* subsp. *spizizenii*, parent strain for mutants	ATCC 6633
DY001 (Δ*glpD*)	6633:: ΔglpD::kan; Km^r	This study
DY002 (Δ*aroB*)	6633:: ΔaroB::kan; Km^r	This study
DY003 (Δ*aroC*)	6633:: ΔaroC::kan; Km^r	This study
DY004 (Δ*aroD*)	6633:: ΔaroD::kan; Km^r	This study
DY005 (Δ*ndh*)	6633:: Δndh::kan; Km^r	This study
DY006 (Δ*ndhF*)	6633:: ΔqoxA::kan; Km^r	This study
DY007 (Δ*qoxA*)	6633:: ΔqoxA::kan; Km^r	This study
DY008 (Δ*cydA*)	6633:: ΔcydA::kan; Km^r	This study
DY009 (Δ*qcrA*)	6633:: ΔqcrA::kan; Km^r	This study
DY010 (Δ*ctaC*)	6633:: ΔctaC::kan; Km^r	This study
BKK09300 (Δ*glpD*)	168::trpC2, ΔglpD::kan; Km^r	Bacillus Genetic Stock Center (BGSC) collection (http://www.bgsc.org/) [27]
BKK22700 (Δ*aroB*)	168::trpC2, ΔaroB::kan; Km^r	
BKK23080 (Δ*aroC*)	168::trpC2, ΔaroC::kan; Km^r	
BKK25660 (Δ*aroD*)	168::trpC2, ΔaroD::kan; Km^r	
BKK12290 (Δ*ndh*)	168::trpC2, Δndh::kan; Km^r	
BKK01830 (Δ*ndhF*)	168::trpC2, ΔndhF::kan; Km^r	
BKK38170 (Δ*qoxA*)	168::trpC2, ΔqoxA::kan; Km^r	
BKK38760 (Δ*cydA*)	168::trpC2, ΔcydA::kan; Km^r	
BKK22560 (Δ*qcrA*)	168::trpC2, ΔqcrA::kan; Km^r	
BKK14890 (Δ*ctaC*)	168::trpC2, ΔctaC::kan; Km^r	

Supplementary Table S4. ***Escherichia coli*** **strains used in this study.**

Escherichia coli **strain**	**Relevant genotype**	**Source or reference**
Escherichia coli MG1655	Parent K-12 strain for mutants, F-, *λ-, ilvG-, rfb-50, rph1*	ATCC 700926
DRK109 (Δ*ubiCA*)	MG1655::*ΔubiCA::kan*; Km^r	26
DRK110 (Δ*ubiE*)	MG1655::*ΔubiE::kan*; Km^r	26
DRK111 (Δ*menA*)	MG1655::*ΔmenA::kan*; Km^r	26
DRK112 (Δ*appB*)	MG1655::*ΔappB::kan*; Km^r	26
DRK113 (Δ*cyoB*)	MG1655::*ΔcyoB::kan*; Km^r	26
DRK114 (Δ*cydB*)	MG1655::*ΔcydB::kan*; Km^r	26
DRK118 (Δ*cyoB*Δ*cydB*)	MG1655::*ΔcyoB::ΔcydB::kan*; Km^r	26
DRK121 (Δ*fdhE*)	MG1655::*ΔfdhE::kan*; Km^r	26
DRK122 (Δ*fdnG*)	MG1655::*ΔfdnG::kan*; Km^r	26
DRK123 (Δ*fdhD*)	MG1655::*ΔfdhD::kan*; Km^r	26
DRK127 (Δ*fdoG*)	MG1655::*ΔfdoG::kan*; Km^r	26
DRK128 (Δ*fdhF*)	MG1655::*ΔfdhF::kan*; Km^r	26
DRK147 (Δ*ubiCA*)	BW25113::*ΔubiCA::kan*; Km^r	26
DRK148 (Δ*menA*)	BW25113::*ΔmenA::kan*; Km^r	26
Escherichia coli BW25113	Parent K-12 strain for Keio collection mutants, *rrnB, DElacZ4787, HsdR514, DE(araBAD)567, DE(rhaBAD)568, rph-1*	Keio collection, National BioResource Project, National Institute of Genetics, Japan [43]
JW3866 (Δ*fdhD*)	BW25113 F-, *Δ(araD-araB)567*, *ΔlacZ4787*(::rrnB-3), λ^-, *rph-1*, *ΔfdhD758::kan*, *Δ(rhaD-rhaB)568*, *hsdR514*	
JW3862 (Δ*fdhE*)	BW25113 F-, *Δ(araD-araB)567*, *ΔlacZ4787*(::rrnB-3), λ^-, *rph-1*, *ΔfdhE754::kan*, *Δ(rhaD-rhaB)568*, *hsdR514*	
JW4040 (Δ*fdhF*)	BW25113 F-, *Δ(araD-araB)567*, *ΔlacZ4787*(::rrnB-3), λ^-, *rph-1*, *Δ(rhaD-rhaB)568*, *ΔfdhF774::kan*, *hsdR514*	
JW3865 (Δ*fdoG*)	BW25113 F-, *Δ(araD-araB)567*, *ΔlacZ4787*(::rrnB-3), λ^-, *rph-1*, *ΔfdoG757::kan*, *Δ(rhaD-rhaB)568*, *hsdR514*	
JW1470 (Δ*fdnG*)	BW25113 F-, *Δ(araD-araB)567*, *ΔlacZ4787*(::rrnB-3), λ^-, *ΔfdnG767::kan*, *rph-1*, *Δ(rhaD-rhaB)568*, *hsdR514*	
JW5581 (Δ*ubiE*)	BW25113 F-, *Δ(araD-araB)567*, *ΔlacZ4787*(::rrnB-3), λ^-, *rph-1*, *ΔubiE778::kan*, *Δ(rhaD-rhaB)568*, *hsdR514*	
JW0421 (Δ*cyoB*)	BW25113 F-, *Δ(araD-araB)567*, *ΔlacZ4787*(::rrnB-3), *ΔcyoB788::kan*, λ^-, *rph-1*, *Δ(rhaD-rhaB)568*, *hsdR514*	
JW0723 (Δ*cydB*)	BW25113 F-, *Δ(araD-araB)567*, *ΔlacZ4787*(::rrnB-3), *ΔcydB782::kan*, λ^-, *rph-1*, *Δ(rhaD-rhaB)568*, *hsdR514*	
JW0961 (Δ*appB*)	BW25113 F-, *Δ(araD-araB)567*, *ΔlacZ4787*(::rrnB-3), λ^-, *ΔappB722::kan*, *rph-1*, *Δ(rhaD-rhaB)568*, *hsdR514*	
JW2280 (Δ*nuoE*)	BW25113 F-, *Δ(araD-araB)567*, *ΔlacZ4787*(::rrnB-3), λ^-, *ΔnuoE767::kan*, *rph-1*, *Δ(rhaD-rhaB)568*, *hsdR514*	
JW2278 (Δ*nuoG*)	BW25113 F-, *Δ(araD-araB)567*, *ΔlacZ4787*(::rrnB-3), λ^-, *ΔnuoG765::kan*, *rph-1*, *Δ(rhaD-rhaB)568*, *hsdR514*	
JW2275 (Δ*nuoJ*)	BW25113 F-, *Δ(araD-araB)567*, *ΔlacZ4787*(::rrnB-3), λ^-, *ΔnuoJ762::kan*, *rph-1*, *Δ(rhaD-rhaB)568*, *hsdR514*	
JW2274 (Δ*nuoK*)	BW25113 F-, *Δ(araD-araB)567*, *ΔlacZ4787*(::rrnB-3), λ^-, *ΔnuoK761::kan*, *rph-1*, *Δ(rhaD-rhaB)568*, *hsdR514*	
JW1095 (Δ*ndh*)	BW25113 F-, *Δ(araD-araB)567*, *ΔlacZ4787*(::rrnB-3), λ^-, *Δndh-771::kan*, *rph-1*, *Δ(rhaD-rhaB)568*, *hsdR514*	

Supplementary Table S5. ***Bacillus subtilis*** **primers used in this study.**

**Primers for Tn-seq**	
oCJ23	AATGATACGGCGACCACCGAGATCTACACGACAGGTTCAGAGTTCTACAGTCCGA
oCJ24	GACAGGTTCAGAGTTCTACAGTCCGACGATCACAC
oCJ25	GTTCAGAGTTCTACAGTCCGACGATCACACNN
oCJ26	PO_4-GTGTGATCGTCGGACTGTAGAACTCTGAACCTGTC-PO_4
oCJ27	ACAGGTTGGATGATAAGTCCCCGGTCTCACACA
oCJ22 BSA-1<br>bar code	CAAGCAGAAGACGGCATACGAGATCGTGATTGTGTGAGACCGGGGACTTATCATCCAACCTGT
oCJ22 PGRP-1<br>17% survival<br>bar code	CAAGCAGAAGACGGCATACGAGATACATCGTGTGTGAGACCGGGGACTTATCATCCAACCTGT
oCJ22 PGRP-1<br>0.5% survival<br>bar code	CAAGCAGAAGACGGCATACGAGATGCCTAATGTGTGAGACCGGGGACTTATCATCCAACCTGT
oCJ22 BSA-2<br>bar code	CAAGCAGAAGACGGCATACGAGATACAGTGTGTGTGAGACCGGGGACTTATCATCCAACCTGT
oCJ22 PGRP-2<br>17% survival<br>bar code	CAAGCAGAAGACGGCATACGAGATGCCAATTGTGTGAGACCGGGGACTTATCATCCAACCTGT
oCJ22 PGRP-2<br>0.5% survival<br>bar code	CAAGCAGAAGACGGCATACGAGATCAGATCTGTGTGAGACCGGGGACTTATCATCCAACCTGT
oCJ22 BSA-3<br>bar code	CAAGCAGAAGACGGCATACGAGATACTTGATGTGTGAGACCGGGGACTTATCATCCAACCTGT
oCJ22 PGRP-3<br>17% survival<br>bar code	CAAGCAGAAGACGGCATACGAGATGATCAGTGTGTGAGACCGGGGACTTATCATCCAACCTGT
oCJ22 PGRP-3<br>0.5% survival<br>bar code	CAAGCAGAAGACGGCATACGAGATTAGCTTTGTGTGAGACCGGGGACTTATCATCCAACCTGT
**Primers for gene deletions**	
glpD	glpD-5pL: 5'-CGATTACTCCAATGCCTCAAG-3'<br>glpD-5pR: 5'-ctctcctttctcgcctgcCATTACGTTTCCTCCTTGTT-3'<br>glpD-3pL: 5'-gcagtgacaggagcctcgTAAATCATAACGGGCTGTCT-3'<br>glpD-3pR: 5'-TGAGAATATCTGCATTCTGCTCC-3'
aroB	aroB-5pL: 5'-CCCAACTGACAACCATTATTGAA-3'<br>aroB -5pR: 5'-ctctcctttctcgcctgcGGTTTGAACATGCAGTGTTT-3'<br>aroB -3pL: 5'-gcagtgacaggagcctcgAAATGGCGATTGGAGGAGAC-3'<br>aroB -3pR: 5'-CTGTTTCTTTTGAATGCGGAG-3'
aroC	aroC-5pL: 5'-ATCGCCTTCATTATTTCTCCTC-3'<br>aroC -5pR: 5'-ctctcctttctcgcctgcCACTCGTCTCAAATCCTTTC-3'<br>aroC -3pL: 5'-gcagtgacaggagcctcgTAGAACAATAAAAAAACTCA-3'<br>aroC-3pR: 5'-ATATCATTACCGTTCACACGCC-3'
aroD	aroD-5pL: 5'-CGATTACTCCAATGCCTCAAG-3'<br>aroD -5pR: 5'-ctctcctttctcgcctgcCATTACGTTTCCTCCTTGTT-3'<br>aroD -3pL: 5'-gcagtgacaggagcctcgTAAATCATAACGGGCTGTCT-3'<br>aroD-3pR: 5'-TGAGAATATCTGCATTCTGCTCC-3'
ndh	ndh-5pL: 5'-CTGCTATTCCTTTCAAATCCG-3'<br>ndh-5pR: 5'-ctctcctttctcgcctgcCATCGTATATCCTCCGTCCT-3'<br>ndh-3pL: 5'-gcagtgacaggagcctcgTAATCCTTTTTGTGAACTCG-3'<br>ndh-3pR: 5'-GCAAAGAAACTGAAGCGTATACGTC-3'
ndhF	ndhF-5pL: 5'-AAACTGGACAGACACCAGCAC-3'<br>ndhF-5pR: 5'-ctctcctttctcgcctgcCATTCTAAATTCTCCCTTTT-3'<br>ndhF-3pL: 5'-gcagtgacaggagcctcgATTTCATAAGGAGCTAATCT-3'<br>ndhF-3pR: 5'-CTCATCAAAGCTGTAGGCAAATG-3'

Supplementary Table S5 continued

qoxA	qoxA-5pL: 5'-ATAAGGAGTGGGCTCTTCGGAT-3'<br>qoxA-5pR: 5'-ctctcctttctcgcctgcCACCATCCTTCCTATTGATG-3'<br>qoxA-3pL: 5'-gcagtgacaggagcctcgTGATATTGATCAGGAAAGGA-3'<br>qoxA-3pR: 5'-CGCCATTGTCGTAATTGAGAAG-3'
cydA	cydA-5pL: 5'-TGCGACAGAACCGGCAATAAGC-3'<br>cydA-5pR: 5'-ctctcctttctcgcctgcCATGCTTTCTCCTCCATTTC-3'<br>cydA-3pL: 5'-gcagtgacaggagcctcgGATCCATTTAGTCAGGAGGT-3'<br>cydA-3pR: 5'-AGTAATCACGGTGAGAATCCGC-3'
qcrA	qcrA-5pL: 5'-AGTGCGTTTGGATCTTGAACTAC-3'<br>qcrA-5pR: 5'-ctctcctttctcgcctgcCATAACTTCTCCCCCCTCTA-3'<br>qcrA-3pL: 5'-gcagtgacaggagcctcgGCAAAGCCTAAGGGGGAAGG-3'<br>qcrA-3pR: 5'-AAAGCGAGTCCCGGCATAATC-3'
ctaC	ctaC-5pL: 5'-ATTCTAATCTCATCACGACTTTCAC-3'<br>ctaC-5pR: 5'-ctctcctttctcgcctgcCATTTCACCCAACCCCTTTT-3'<br>ctaC-3pL: 5'-gcagtgacaggagcctcgTAAATCATAACGGGCTGTCT-3'<br>ctaC-3pR: 5'-TGAGAATATCTGCATTCTGCTCC-3'
Kanamycin-specific primers	Kan F 5'- GCAGGCGAGAAAGGAGAGANNCNNCNNGNNANNGNNCNNGAGGGAGGAA AGGCAGGA -3'<br>Kan R 5'- CGAGGCTCCTGTCACTGCNNGNNCNNTNNGNNCNNTNNGCGCCGTATCTGT GCTCTC -3'
**Kanamycin and gene 3' flanking region primers for verification of deletions by PCR**	
Kanamycin	Kan-2 F 5'- AGTAAGTGGCTTTATTGATCTTGGG -3'
glpD	glpD-3pR: 5'-TGAGAATATCTGCATTCTGCTCC-3'
aroB	aroB -3pR: 5'-CTGTTTCTTTTGAATGCGGAG-3'
aroC	aroC-3pR: 5'-ATATCATTACCGTTCACACGCC-3'
aroD	aroD-3pR: 5'-TGAGAATATCTGCATTCTGCTCC-3'
ndh	ndh-3pR: 5'-GCAAAGAAACTGAAGCGTATACGTC-3'
ndhF	ndhF-3pR: 5'-CTCATCAAAGCTGTAGGCAAATG-3'
qoxA	qoxA-3pR: 5'-CGCCATTGTCGTAATTGAGAAG-3'
cydA	cydA-3pR: 5'-AGTAATCACGGTGAGAATCCGC-3'
qcrA	qcrA-3pR: 5'-AAAGCGAGTCCCGGCATAATC-3'
ctaC	ctaC-3pR: 5'-TGAGAATATCTGCATTCTGCTCC-3'
**Coding region gene-specific primers for verification of deletions by PCR**	
glpD	glpD-5': TTATTGCTCAAGCGGTACGA<br>glpD-3': ATGATGAATCATCAATTTTC
aroB	aroB-5': ATTGATATCTTCCTCCGTTATGTAAA<br>aroB-3': GAGCTGTCGGGTACTTAAGCTACGA
aroC	aroC-5': GTGAACGTGTTAACGATTAA<br>aroC-3': CTATCCCCGTGTGTTTTTAT
aroD	aroD-5': TCTTTCGTAAGCGATGCAGGCGGCG<br>aroD-3': GATGAACACGGCGATCGAACTTGCG
ndh	ndh-5': AGCTTTTTATGGCCAACTCCGGCTG<br>ndh-3': ACGGATTTTTTCTGACAGATTACAAG
ndhF	ndhF-5': ATGTTAGTTTCGCTGAGTTT<br>ndhF-3': TTATGAAATATATTGTTTAA
qoxA	qoxA-5': TCATTCTTCTGTATCATCAG<br>qoxA-3': GTGATCTTCTTGTTCAGAGC
cydA	cydA-5': ATGAGTGAATTGGTATTAGC<br>cydA-3': TCATGAAGAGATGCCATGGT
qcrA	qcrA-5': TCACCCTTCCCCCTTAGGCT<br>qcrA-3': ATGGGCGGAAAACATGATAT
ctaC	ctaC-5': ATGGTAAAGCATTGGCGTCT<br>ctaC-3': CTACTTGCTTTCCGCTTTTA
